# Acceptor Specificity of β-*N*-Acetylhexosaminidase from *Talaromyces flavus*: A Rational Explanation

**DOI:** 10.3390/ijms20246181

**Published:** 2019-12-07

**Authors:** Cecilia Garcia-Oliva, Pilar Hoyos, Lucie Petrásková, Natalia Kulik, Helena Pelantová, Alfredo H. Cabanillas, Ángel Rumbero, Vladimír Křen, María J. Hernáiz, Pavla Bojarová

**Affiliations:** 1 Department of Chemistry in Pharmaceutical Sciences, Faculty of Pharmacy, Complutense University of Madrid, Plaza Ramón y Cajal, E 28040 Madrid, Spain; cecilag@ucm.es (C.G.-O.); pilarhv@farm.ucm.es (P.H.); 2 Institute of Microbiology of the Czech Academy of Sciences, Vídeňská 1083, CZ 14220 Prague 4, Czech Republic; petraskova@biomed.cas.cz (L.P.); pelantova@biomed.cas.cz (H.P.); kren@biomed.cas.cz (V.K.); 3 Institute of Microbiology of the Czech Academy of Sciences, Center for Nanobiology and Structural Biology, Zámek 136, CZ 37333 Nové Hrady, Czech Republic; kulik@nh.cas.cz; 4 Department of Organic Chemistry, Autonomous University of Madrid, Cantoblanco, 28049 Madrid, Spain; mjhernai@farm.ucm.es (A.H.C.); angel.rumbero@uam.es (Á.R.); 5 Department of Health Care Disciplines and Population Protection, Faculty of Biomedical Engineering, Czech Technical University in Prague, Nám. Sítná 3105, CZ 27201 Kladno, Czech Republic

**Keywords:** β-*N*-acetylhexosaminidases, substrate specificity, transglycosylation, Glide docking, *Talaromyces flavus*, muramic acid, non-reducing carbohydrate

## Abstract

Fungal β-*N*-acetylhexosaminidases, though hydrolytic enzymes in vivo, are useful tools in the preparation of oligosaccharides of biological interest. The β-*N*-acetylhexosaminidase from *Talaromyces flavus* is remarkable in terms of its synthetic potential, broad substrate specificity, and tolerance to substrate modifications. It can be heterologously produced in *Pichia pastoris* in a high yield. The mutation of the Tyr470 residue to histidine greatly enhances its transglycosylation capability. The aim of this work was to identify the structural requirements of this model β-*N*-acetylhexosaminidase for its transglycosylation acceptors and formulate a structure–activity relationship study. Enzymatic reactions were performed using an activated glycosyl donor, 4-nitrophenyl *N*-acetyl-β-d-glucosaminide or 4-nitrophenyl *N*-acetyl-β-d-galactosaminide, and a panel of glycosyl acceptors of varying structural features (*N*-acetylglucosamine, glucose, *N*-acetylgalactosamine, galactose, *N*-acetylmuramic acid, and glucuronic acid). The transglycosylation products were isolated and structurally characterized. The C-2 *N*-acetamido group in the acceptor molecule was found to be essential for recognition by the enzyme. The presence of the C-2 hydroxyl moiety strongly hindered the normal course of transglycosylation, yielding unique non-reducing disaccharides in a low yield. Moreover, whereas the *gluco*-configuration at C-4 steered the glycosylation into the β(1-4) position, the *galacto*-acceptor afforded a β(1-6) glycosidic linkage. The Y470H mutant enzyme was tested with acceptors based on β-glycosides of uronic acid and *N*-acetylmuramic acid. With the latter acceptor, we were able to isolate and characterize one glycosylation product in a low yield. To our knowledge, this is the first example of enzymatic glycosylation of an *N*-acetylmuramic acid derivative. In order to explain these findings and predict enzyme behavior, a modeling study was accomplished that correlated with the acquired experimental data.

## 1. Introduction

The study of carbohydrates is essential to understand their role in the diverse biological activities in which they occur, mainly as part of glycoconjugates. Inflammation, coagulation, metastasis, immunomodulation, bacterial and viral infection are some of the processes which can be understood in depth through the synthesis of glycomimetics [1,2,3,4].

β-*N*-Acetylhexosaminidases (EC 3.2.1.52, CAZy GH20) are known in nature for their hydrolytic activity. They catalyze the cleavage of the non-reducing end of oligosaccharide chains containing *N*-acetyl-β-d-glucosaminide (GlcNAc) or *N*-acetyl-β-d-galactosaminide (GalNAc) units. However, in vitro, the reaction equilibrium can be shifted in favor of transglycosylation by changing the kinetic conditions (activated glycosyl donor, donor/acceptor ratio) and/ or by mutating the enzyme in the active site [5,6].

The broad range of substrates accepted makes these enzymes ideal synthetic tools with non-natural substrates [7]. In this context, β-*N*-acetylhexosaminidases from fungi have been demonstrated as attractive catalysts for this purpose [8,9,10]. Moreover, genetic engineering has enabled the modification of these enzymes, making them more convenient for the synthesis and expanding the spectrum of identified substrates [11]. 

The standard reaction used to test the transglycosylation performance of β-*N*-acetylhexosaminidases involves the use of an activated glycosyl donor such as *p*NP-GlcNAc or *p*NP-GalNAc and a glycosyl acceptor, for example, GlcNAc. The ratio between the donor and acceptor substrates must be low enough to promote the synthesis and minimize the hydrolysis. Chitooligosaccharides, the natural substrates of β-*N*-acetylhexosaminidases, consist of subunits linked by a β(1-4) glycosidic bond, which is thus the preferred linkage when oligosaccharide chains are formed [12]. β-*N*-Acetylhexosaminidases utilize a specific type of retaining catalytic mechanism of glycosidases, termed substrate-assisted catalysis. It is typical of the formation of an oxazolinium reaction intermediate (Supplementary Materials, Scheme S1).

In this work, we have focused on the β-*N*-acetylhexosaminidase from *Talaromyces flavus* CCF 2686 (*Tf*Hex), a representative of transglycosylating β-*N*-acetylhexosaminidases, due to its great substrate flexibility, which enhances its potential in synthetic reactions, and the availability of the molecular model of this enzyme. In addition, site-directed mutagenesis has allowed the improvement of its synthetic properties, generating a considerable increase in its transglycosylation capability through the substitution of the enzyme active site residue tyrosine 470 by histidine [11,13]. The *Tf*Hex Y470H mutant has been demonstrated as an efficient synthetic tool [14]. When quantifying its hydrolytic activity, the wild-type enzyme (*Tf*Hex WT) is ca. 200 times more efficient than *Tf*Hex Y470H. While *Tf*Hex WT is more active when hydrolyzing *p*NP-GalNAc, *Tf*Hex Y470H has a preference for the *pNP*-GlcNAc donor substrate.

So far, the acceptor specificity of these enzymes has not been thoroughly analyzed since previous studies have mainly focused on donor recognition [8,15]. Therefore, this work aims to explore the synthetic potential of *Tf*Hex WT and *Tf*Hex Y470H with a range of acceptor substrates, by gathering the structural requirements of the enzyme and exploring the spectrum of feasible reactions. In the frame of this study, a variety of natural monosaccharides derived from a *gluco*- or *galacto*-configuration were selected as acceptors: *N*-acetylglucosamine (GlcNAc; **1**), glucose (Glc; **2**), *N*-acetylmuramic acid (MurNAc; **3**), glucuronic acid (GlcA; **4**), galactose (Gal; **6**), and *N*-acetylgalactosamine (GalNAc; **5**), as well as other non-natural glycosides of *N*-acetylmuramic acid and glucuronic acid, functionalized at the anomeric position [16]. These compounds share a similar structure, but differ in the position or presence of some substituents. Through this strategy, we aimed to deduce which structural moieties in the acceptors with the *gluco*- or *galacto*-core are essential for the recognition by *Tf*Hex. A detailed molecular modeling study was performed to understand the enzyme behavior and determine the key residues participating in the interaction.

## 2. Results

### 2.1. Screening of the Glycosylation of Various Acceptors

#### 2.1.1. Glycosylation Reaction Catalyzed by *Tf*Hex WT: Screening of Natural Monosaccharides

**Figure 1:**
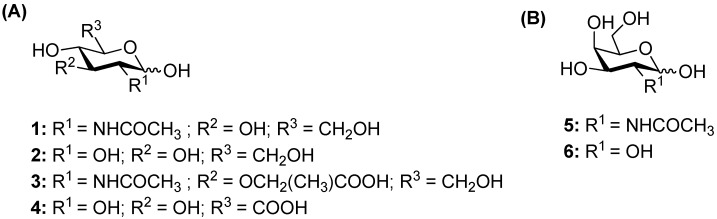
Selection of acceptors screened with the wild-type enzyme (*Tf*Hex WT). (**A**) Acceptors based on the *gluco*-configuration: *N*-acetylglucosamine (**1**), glucose (**2**), *N*-acetylmuramic acid (**3**), and glucuronic acid (**4**); (**B**) acceptors based on the *galacto*-configuration: galactose (**6**) and *N*-acetylgalactosamine (**5**).

As exemplified above, *Tf*Hex WT is an excellent tool in carbohydrate synthesis [7]. For the aim of exploring the spectrum of acceptor substrates of this enzyme, we first decided to test its activity in the transglycosylation reaction involving *p*NP-GlcNAc or *p*NP-GalNAc as glycosyl donors and various natural monosaccharides of the *gluco*- and *galacto*-configuration as acceptors, namely GlcNAc (**1**), Glc (**2**), MurNAc (**3**), GlcA (**4**), Gal (**6**), and GalNAc (**5**) (Figure 1). As GlcNAc is the natural acceptor substrate of this enzyme, the transglycosylation reaction employing *p*NP-GlcNAc and GlcNAc (**1**) was used as a reference reaction in order to optimize the enzyme amount and concentration of donor and acceptor substrates. The donor/acceptor ratio of 1/6 (50 mM donor and 300 mM acceptor) was found as the optimum to shift the equilibrium towards the formation of the transglycosylation product. First, reactions with acceptor substrates **1**–**6** were conducted at an analytical scale (200 μL) and in case product formation was detected, reactions were scaled up to a 3 mL volume. The outcome of respective transglycosylation reactions is shown in Figures S1–S6 in the Supplementary Materials.

**Figure 2:**
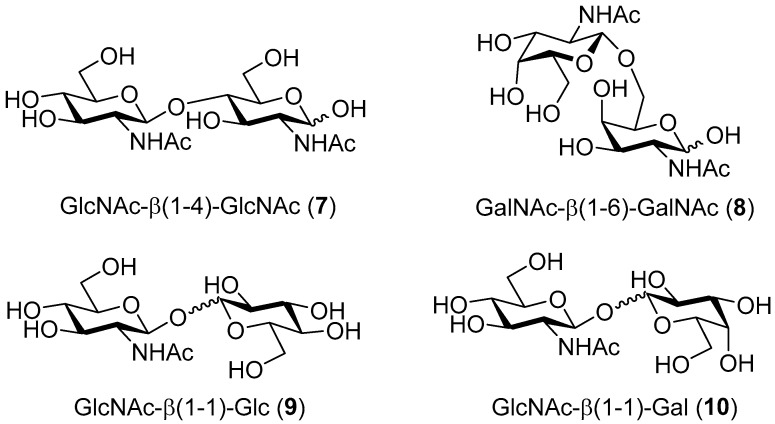
Products of the glycosylation of GlcNAc (**1**), GalNAc (**5**), glucose (**2**), and galactose (**6**) catalyzed by *Tf*Hex WT.

The reference reaction between *p*NP-GlcNAc and GlcNAc (**1**) (Table 1, entry I), selectively afforded the corresponding *N*,*N*′-diacetylchitobiose product GlcNAc-β(1-4)-GlcNAc (**7**, Figure 2) in a 24% yield. In contrast, the reaction performed with the *p*NP-GalNAc donor and GalNAc (**5**) acceptor (Table 1, entry II), exclusively resulted in the β(1-6) regioisomer (**8**, Figure 2) in a 31% yield. As a rule of thumb, the axial *galacto*-position is considered practically impossible to glycosylate with β-*N*-acetylhexosaminidases [7]. This study demonstrates the high regioselectivity of β-*N*-acetylhexosaminidase-catalyzed glycosylation, depending on the configuration of the C-4 acceptor hydroxyl (Figure 2).

When glucose (**2**) and galactose (**6**), lacking the C-2 *N*-acetamido group, were tested as acceptors (Table 1, entries III and IV, respectively), a small amount of the transglycosylation product was detected by thin layer chromatography (TLC) (Figures S3 and S4, Supplementary Material). After isolation by gel chromatography, the formation of a mixture of disaccharides was found by NMR spectroscopy, with the respective non-reducing β(1-1) disaccharide being among the major identified products (Figure 2, compounds **9** and **10**, respectively). The unexpected finding of non-reducing disaccharides as products of glycosylation of both glucose and galactose highlights the vital role of the *N*-acetamido group, not only for substrate recognition, but also for favorable acceptor orientation in the active site of the *Tf*Hex WT enzyme.

MurNAc (**3**) and GlcA (**4**) were also tested as challenging acceptors in the transglycosylation reactions mediated by *Tf*Hex WT. We speculated whether carboxy groups of MurNAc (**3**) and GlcA (**4**) may interfere in the accommodation of the compounds in the active site due to their negative charge. Therefore, in order to avoid the repulsion between the acceptor carboxy group and the charged residues in the enzyme’s active site, methyl esters of **3** and **4** were first synthesized and tested as acceptors. However, the esters proved to be unstable under reaction and purification conditions, and were spontaneously hydrolyzed, affording the acidic form. Therefore, monosaccharides **3** and **4** with free carboxy groups were tested for glycosylation. In both cases, synthesis of the expected disaccharides failed and only the hydrolytic product, GlcNAc, could be detected, even when different fractions were analyzed after purification.

#### 2.1.2. Glycosylation Reaction Catalyzed by TfHex Y470H: Screening of GlcA and MurNAc Glycosides

**Figure 3:**
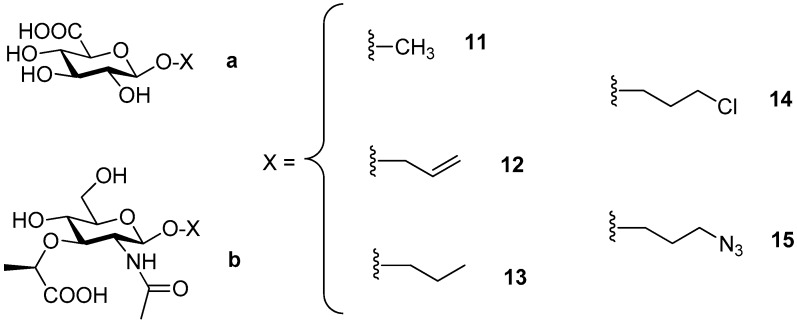
Series of β-glycosides of GlcA (**11a**–**15a**) and MurNAc (**11b**–**15b**) modified at the anomeric position.

Since GlcA (**4**) and MurNAc (**3**) were not recognized as acceptors by the wild-type enzyme, they were subjected to testing using the mutant enzyme *Tf*Hex Y470H with suppressed hydrolytic activity and increased transglycosylation potential [13]. Besides free monosaccharides, a series of GlcA and MurNAc glycosides [16] functionalized at the anomeric position (**11**–**15**, Figure 3) were also employed as acceptors in the transglycosylation reaction mediated by this biocatalyst to test the influence of the anomeric effect on the glycosylation process. GlcA (**11a**–**15a**) and MurNAc glycosides (**11b**–**15b**) were prepared according to the protocol previously described [16].

**Figure 4:**
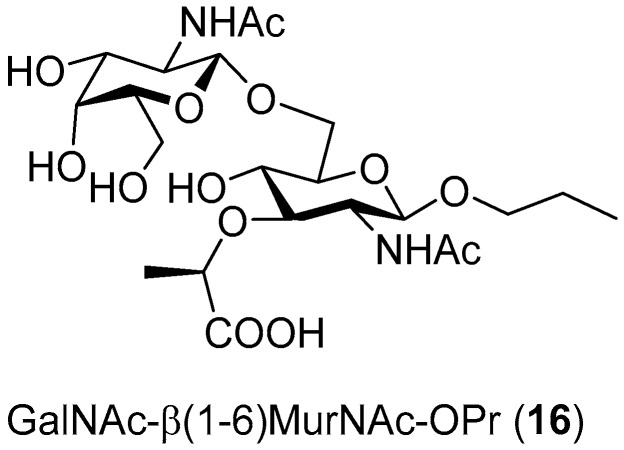
Disaccharide **16** synthesized by the transglycosylation of MurA glycoside (MurNAc-*O*Pr) **13b** with GalNAc under the catalysis by *Tf*Hex Y470H.

When these acceptors were screened in transglycosylation reactions using *p*NP-GlcNAc as a donor, a range of chitooligomers of different lengths were detected in the reaction, which is typical of this mutant enzyme, as reported previously [11]. Therefore, it seemed that *p*NP-GalNAc may be the donor of preference since the formation of longer oligosaccharides was not observed with this mutant enzyme [14]. In accord with our previous experience, the optimum donor/acceptor ratio was higher thanks to the increased synthetic potential of the mutant enzyme and for the sake of minimizing the consumption of the acceptor employed. The most promising reactions, based on TLC and ESI-MS results, which were scaled-up, are summarized in Table 1. Unfortunately, no products were detected with either GlcA (**4**) or GlcA glycosides (**11a**–**15a**). In contrast, MurNAc (**3**) seemed to be better recognized by the enzyme. In the reaction with the *p*NP-GlcNAc donor and MurNAc acceptor, the expected product was detected by ESI-MS after 7 h. Regrettably, we failed to purify it to the extent to be analyzed by NMR in order to determine the regioselectivity of the reaction. In the screening of MurNAc glycosides (**11b**–**15b**), the best result was achieved when MurNAc-*O*Pr (**13b**) was employed as an acceptor and *p*NP-GalNAc as a donor, affording the corresponding β(1-6) regioisomer **16** (Figure 4), though in a low yield (ca. 1%). We speculate that the presence of the lactate ether group at C-3 apparently impaired recognition by the enzyme, resulting in an unfavorable orientation for glycosylation. We further hypothesize that the presence of the bulky lactate ether promoted the glycosylation to the β(1-6) position, despite the otherwise strong enzyme selectivity for the β(1-4) bond in the case of GlcNAc. The low yield may also have been partially caused by the complex purification comprising gel permeation chromatography and silica gel chromatography.

### 2.2. Docking and Molecular Dynamics 

#### 2.2.1. Docking and Molecular Dynamics Simulation of Selected Transglycosylation Acceptors in *Tf*Hex WT 

To investigate the structural properties of *Tf*Hex and its ability to catalyze glycosidic bond formation, we performed the docking of transglycosylation acceptors in the active site of equilibrated complexes of WT and Tyr470His *Tf*Hex with GlcNAc-oxazoline (GlcNAcox) and GalNAc-oxazoline (GalNAcox) as mimics of intermediates during the hydrolysis of respective donors (*p*NP-GlcNAc or *p*NP-GalNAc). Interactions of GlcNAcox and GalNAcox intermediates with respective active site amino acid residues are shown in Figure S7 (Supplementary Materials). For equilibration parameters, see Figures S8 and S9 (Supplementary Materials). Scaled binding scores of transglycosylation acceptors are shown in Table 2.

There is an alternative binding place close to the active site with bound oxazoline intermediates, which is available for all acceptors—it is denoted here as site 1. In the WT enzyme, this site is more accessible for smaller ligands (Gal, Glc, GalNAc, GlcNAc) than for bulkier ones. Site 1 is placed close to the active site (Figure S10, Supplementary Materials) and is surrounded by residues Asn322, Glu332, Gly476, Gly477, Phe478, Arg484, Gly507, Trp509, Glu546, and Gln547, many of which belong to the flexible loops framing the active site [17]. This binding site is relatively well-accessible and is not a randomly formed temporary structure, as assumed from the ability of acceptors to dock in site 1 (Table 2) and from their stability in this position during molecular dynamics (MD) simulations (Figure S10, Supplementary Materials). However, acceptor binding in this site disables the productive outcome of the transglycosylation reaction due to a mutual unfavorable orientation of the donor and acceptor. Another alternative binding site—site 2—directs the binding of acceptors mostly above the donor, again with orientations unfavorable for the transglycosylation reaction— too far from the anomeric carbon of donor, too far from the catalytic base Glu371, or too far from both. This site is an unspecific binding site, the interactions of which greatly differ among individual acceptors. In Table 2, the affinity of both of these alternative binding sites to respective acceptors is presented with a binding score. 

**Figure 5:**
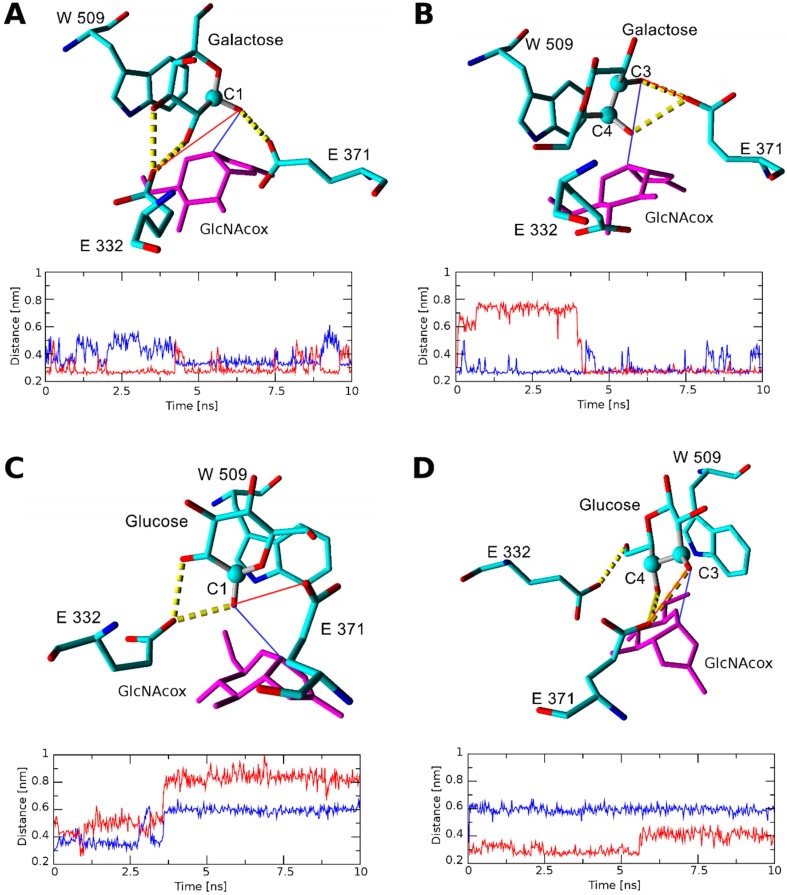
Interaction of glucose and galactose docked in the WT-GlcNAcox complex after 10 ns of molecular dynamics. Hydrogen bonds (HB) are shown by yellow dashed lines. Acceptor atoms docked close to Glu371 at the beginning of the molecular dynamics simulation are represented by a ball. (**A**) Snapshot of the WT-GlcNAcox complex with galactose docked with C-1 close to Glu371, and the distance between O-1 of Gal and the OE2 atom of Glu371 in (**red**) or C-1 of GlcNAcox (**blue**). The respective distances are indicated in the models in respective colors. (**B**) Snapshot of the WT-GlcNAcox complex with galactose docked with C-3 close to Glu371 and the distance between O-3 of Gal and the OE2 atom of Glu371 (**red**) or C-1 of GlcNAcox (**blue**). The respective distances are indicated in the models in respective colors. (**C**) Snapshot of the WT-GlcNAcox complex with glucose docked with C-1 close to Glu 371, and the distance between C-1 of GlcNAcox (**blue**) or the OE2 atom of Glu371 (**red**) and O-1 of glucose. The respective distances are indicated in the models in respective colors. (**D**) Snapshot of the WT-GlcNAcox complex with glucose docked with C-3 close to Glu371, and the distance between C-1 of GlcNAcox (**blue**) or the OE1 atom of Glu371 (**red**), and O-3 of glucose. The respective distances are indicated in the models in respective colors.

The binding score for the galactose acceptor in all found orientations with *Tf*Hex WT was the highest of all acceptors examined, so galactose may be considered the worst acceptor of the series. The most favorable binding scores were found for β(1-3) bond formation and for binding in site 1. To analyze the stability of docked galactose, we performed short molecular dynamics simulations, which revealed that in the case of β(1-3) binding, galactose is rotated unfavorably for product formation and the C-3 atom moves much further from the transglycosylation donor (Figure 5). Finally, during molecular dynamics with galactose, the catalytic Glu371 forms hydrogen bonds (HB) with C-3 and C-4 hydroxyls, which influences the charge distribution in the catalytic base Glu371 and complicates the involvement of respective hydroxyls in the formation of the glycosidic bond. Notably, in the case of galactose docked with C-1 close to Glu371, a close interaction with both Glu371 and the transglycosylation donor is preserved during a long molecular dynamics simulation time (Figure 5A). This could explain the enzyme preference for β(1-1) product formation. The ability of galactose to bind in site 1 with a similar score demonstrates a low specificity of binding and, together with the high value of the binding score, accounts for a low transglycosylation yield. Similarly, in the case of glucose, the binding in the WT-GlcNAcox system could lead to many products; the best binding scores are found for site 1, and for positions C-3 and C-4 close to catalytic Glu371. Orientation with the formation of the β(1–3) glycosidic bond is excluded for glucose as well as for galactose, as proved by molecular dynamics (Figure 5D) due to the acceptor rotation. In the *Tf*Hex WT-GalNAcox complex, galactose and glucose are not bound properly for productive transglycosylation due to the axial orientation of the C-4 hydroxyl of GalNAc oxazoline. 

**Figure 6:**
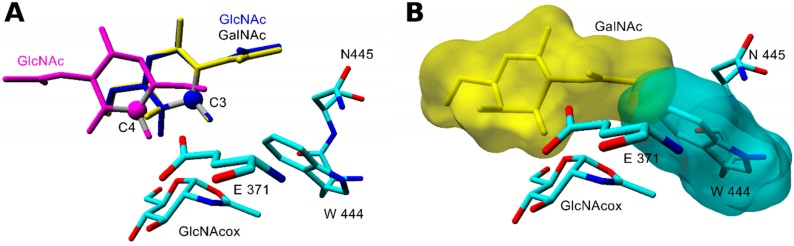
Steric conflict in the formation of the transglycosylation product with the β(1-3) glycosidic bond. (**A**). Overlay of docked orientations of GlcNAc (**blue**) and GalNAc (**yellow**) acceptors with C-3 or C-4 (GlcNAc in **magenta**) close to Glu371 in the WT-GlcNAcox complex. Active site residues within 3 Å from the GalNAc *N*-acetyl group are shown. (**A**) change that would be required in the C-3 position for product formation is indicated by the red dotted arrow. (**B**) Expected orientation of the GalNAc acceptor needed for the formation of GalNAc-β(1-3)-GlcNAc in the active site of WT-GlcNAcox. Position of the acceptor is determined from the alignment with *N*,*N*′-diacetylchitobiose (PDB ID: 1qbb). Intersection of the molecular surfaces of GalNAc (**yellow**) and Trp444 (**cyan**) shows that the GalNAc acceptor cannot move closer to the transglycosylation donor for the product formation due to steric hindrance.

In the case of the GalNAc acceptor, the preferred orientation is with its C-3 hydroxyl close to the carboxyl of catalytic Glu371. However, the respective product with the β(1-3) bond cannot be formed due to the steric conflict between the *N*-acetyl group and Trp444 (Figure 6). Importantly, the inability to form the β(1-3) bond due to steric reasons may relate to all acceptors with *N*-acetyl substitution at C-2, namely GlcNAc, GalNAc, MurNAc, and MurNAc-*O*Pr. This inability is due to the unfavorable orientation of the *N*-acetyl group above hydrophobic Trp444. Concerning the GlcNAc acceptor docked with C-4 close to Glu371, its HB with Glu371, Glu332, and the backbone atoms of Val331 (interacting with the *N*-acetyl group) improves its stability in the active site. A favorable and stable interaction with Glu371 and GlcNAc oxazoline was found until 6.5 ns of MD. The binding scores with other possible orientations (C-3, C-6, and site 2) were twice as bad. This may account for a high yield and good selectivity of the β(1-4) product with GlcNAc (see also Figure S11, Supplementary Materials). GlcNAc can bind in a similar orientation to the WT-GalNAcox complex but with a lower productivity since it has a better binding score for site 1. 

With the GalNAc acceptor, the situation is similar. It binds with a lower productivity to the complex with GalNAc oxazoline (higher binding score for site 1), and the β(1-3) product formation is sterically hampered by Trp444 (Figure 6). MD simulation also confirmed a fast change of GalNAc acceptor orientation with water penetration close to Glu371 (data not shown). With both GalNAc and GlcNAc acceptors, the binding scores of β(1-6) bond formation are energetically less favorable. The orientation of acceptors with their C-6 close to C-1 of WT-GlcNAcox was unstable during molecular dynamics and resulted in a fast increase of this distance (data are not shown). However, due to the hindrance of β(1-3) bond formation, for the GalNAc acceptor, the β(1-6) bond is still the most feasible possibility as also demonstrated in the synthetic experiment. 

**Figure 7:**
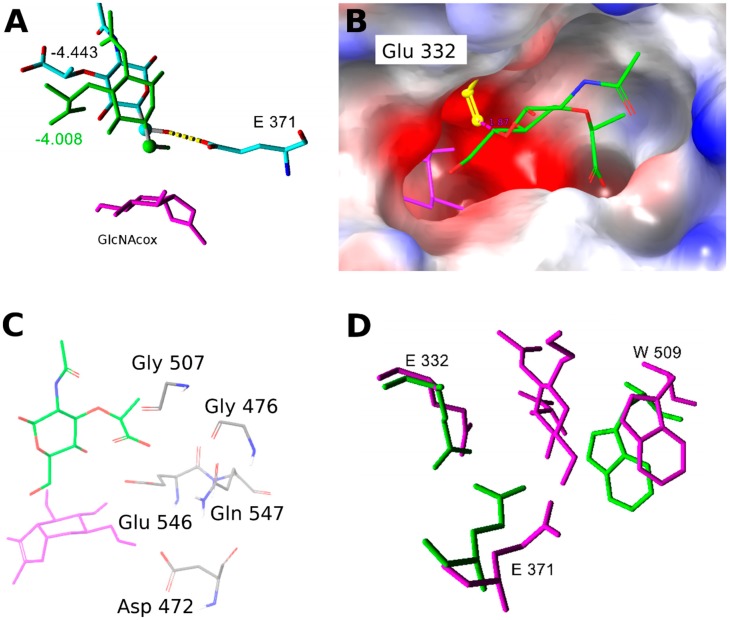
(**A**) Orientation of MurNAc in the WT-GlcNAcox complex: **green**—close to the transglycosylation donor; **element** color—far, corresponds to site 2. The respective Glide absolute binding scores are shown in the figure. GlcNAcox is shown in **magenta**, hydrogens are hidden, and the C-6 atom is represented by a ball. (**B**) Electrostatic potential surface of the WT enzyme active site with GlcNAcox (**magenta**) and the MurNAc acceptor (**element** color). Orientation of Glu332 (**yellow**) in one of the analyzed snapshots disallows a close interaction between the acceptor and donor. The negatively charged environment is shown in **red**, and that which is positively charged is presented in **blue**. (**C**) Residues with a negative charge in the vicinity of the MurNAc acceptor carboxyl group when it binds close to the transglycosylation donor. (**D**) Overlay of the aglycone binding site residues of WT-GlcNAcox (**green**) and Y470H-GalNAcox (**magenta**) with docked MurNAc-*O*Pr. Residues with similar side chain orientations are hidden.

The acceptors containing a carboxy group may exist in various protonation states; p*K*_a_ of GlcA varies from 2.83 to 3.28. Therefore, for the experimental situation at pH 5 used in this study, we modeled the respective deprotonated forms as prevalent under the transglycosylation conditions. In GlcA, the preferred orientation corresponds to acceptor binding in sites 1 or 2. In the case of MurNAc and MurNAc-*O*Pr in the WT-GlcNAcox complex, binding prevails at site 2, but C-6 is often close to Glu371 (Figure 7). Surprisingly, site 1 could not be targeted with a favorable score. There, a closer positioning of C-6 of MurNAc and MurNAc-*O*Pr was hindered by Glu332 rotation (Figure 7B). Another reason for the worse binding of MurNAc and MurNAc-*O*Pr of the WT-GlcNAcox complex is electrostatic—there are negatively charged residues in the vicinity of the acceptor carboxyl group when it binds closer to the transglycosylation donor (Figure 7 and Figure S12). The amino acids in the vicinity of the active site disallow C-6 hydroxyl to come closer to the transglycosylation donor and also restrict the conformational flexibility of MurNAc and MurNAc-*O*Pr acceptors. In the WT-GalNAcox complex, GlcA and MurNAc acceptors clearly bind best in the unproductive sites 1 or 2. Other orientations have more than 1 kcal/mol higher binding scores. During the molecular dynamics simulation of MurNAc in WT-GlcNAcox, the acceptor could approach GlcNAcox closer, but Glu371 was moved far from it and the active site was slightly destroyed. Additionally, due to the orientation of MurNAc, many water molecules come into the active site close to Glu371. In contrast, the MurNAc-*O*Pr acceptor maintained a favorable position in the active site during molecular dynamics and interactions with Glu371 and GlcNAcox were stable with low distances (data not shown). The docked orientation of MurNAc-*O*Pr in the Y470H mutant enzyme is slightly different (Figure S13), leading to a different transition state in the glycosylation step that may influence the product formation. Aliphatic propyl at C-1 may also reduce water access to the active site. 

#### 2.2.2. Docking and Molecular Dynamics Simulation of Selected Transglycosylation Acceptors in *Tf*Hex Y470H

The mutant *Tf*Hex Y470H, previously demonstrated as an efficient synthetic tool [13,14], was selected for the study with difficult acceptors GlcA, MurNAc, and MurNAc-*O*Pr. All acceptors docked in the respective enzyme-oxazoline complexes showed unfavorable scores for binding, with the most favorable poses in sites 1 or 2. The Y470H-GalNAcox complex showed a low affinity to all studied acceptors, except for MurNAc-*O*Pr. We compared amino acid residues close to docked MurNAc-*O*Pr (Figure 7D) in the WT and mutant enzymes and found that residues Trp509 and Glu332 of the aglycone biding site have different side chain orientations, and thus influence the size of the aglycone binding site. The mutation of Tyr470 to His led to the loss of HB with the oxazoline intermediate and to the formation of different HB interactions with residues close to Trp509 (Supplementary Materials, Figure S7, and [13]). The Y470H mutant differs from the WT enzyme not only in the orientation of residues in the active site but also in the position of the oxazoline intermediate (Figure S7). The binding pocket surface, calculated for representative snapshots from molecular dynamics by Sitemap, showed a larger aglycone binding site surface for the Y470H-GalNAcox complex (1204.83 sq. Å) whereas for other combinations, it was significantly smaller (*cf*. 1003.468 sq. Å for WT-GalNAcox; 1094.485 sq. Å for Y470H-GlcNAcox; 839.991 sq. Å for WT-GlcNAcox).

## 3. Discussion

In this study, we aimed to thoroughly understand the performance of *Tf*Hex as a representative transglycosylating β-*N*-acetylhexosaminidase in terms of acceptor recognition. Therefore, we employed a series of acceptors with varying structural features based on *gluco*- or *galacto*-configuration. The enzyme tolerance to structural modifications in the donor molecule has been studied at great length [7]; however, little is known about the acceptor spectrum. *Tf*Hex has been known as an enzyme strictly selective for β(1-4) bond formation and a simplistic approach would suggest that if the activated donor is cleaved and an excess acceptor is present, disaccharide formation is nearly guaranteed. In this study, we demonstrate that the issue is far more complex and that the prediction of acceptor substrate specificity is even more challenging than with glycosyl donors. Docking allows us to identify possible binding poses of transglycosylation acceptors and reveal residues participating in the interaction. However, the number of expected products based on docking results is much higher than in the experiment. It is related to the uncertainty in the selection of an apt threshold for binders and of parameters for the validation of binding poses. Moreover, it appears that even a single point mutation in the active site may influence the enzyme behavior in far more aspects than supposed at first sight, as demonstrated in the example of the Y470H mutant.

Therefore, what structural aspects are critical for acceptor recognition and a good transglycosylation outcome? The first and foremost conclusion of this study is the vital importance of the C-2 *N*-acetamido moiety in the acceptor molecule. Whereas both GlcNAc and GalNAc acceptors are readily selectively glycosylated, the situation in the case of their structural counterparts Glc and Gal is drastically different. Not only is their glycosylation very poor, but it lacks selectivity and yields peculiar non-reducing disaccharides. This type of bond, where both reducing ends are implicated, has already been reported, to a lesser extent, during which non-natural substrates were employed with β-*N*-acetylhexosaminidases [18]. The theory highlighting the importance of the *N*-acetyl group at acceptor C-2 is further supported by the example of GlcA. Our previous experience showed that uronic acid of *p*NP-GlcNAc is readily hydrolyzed and subject to auto-condensation, forming the respective disaccharide [8]. However, with the GlcA acceptor void of an *N*-acetamido moiety, no disaccharide could be detected, irrespective of any substitution at C-1. Scaled docking binding scores allowed us to identify many possible orientations of transglycosylation acceptors, while further molecular dynamics refinement and analysis were needed to filter out false positive results.

The present results strongly support the rule of thumb on the inability of β-*N*-acetylhexosaminidases to glycosylate axial hydroxyls. Despite its high selectivity for the β(1-4) bond, the GalNAc acceptor was willingly glycosylated at C-6 with a high selectivity. In accordance with previous results, the β(1-3) bond was not detected in any case. As nicely explained by the docking studies, the formation of the β(1-3) linkage is hampered by the position of the substrate *N*-acetyl group in the enzyme active site.

The third main contribution of this study is the first ever example of the glycosylation of a challenging acceptor of MurNAc by a glycosidase, though with a low yield. Even here, the presence of a rather bulky substituent at C-3 has consequences for the reaction regioselectivity—in spite of the presence of the C-2 *N*-acetamido group, MurNAc is a very difficult acceptor, directing glycosylation regioselectivity to the β(1-6) position. Apparently, the presence of a larger substituent at C-1, such as propyl, enhanced the recognition of the substrate by the enzyme, being even more favorable in the Y470H mutant than in the WT. The seminal role of propyl may consist in reducing the access of water to the active site. This is because the different orientation of Glu332 and Trp509 regulates the size of the aglycone binding site and, together with GalNAcox, influences the mutant enzyme affinity to MurNAc-*O*Pr. Disaccharide GalNAc-β(1-6)-MurNAc-*O*Pr is the first enzymatically glycosylated MurNAc derivative and it opens new possibilities in enzymatic synthesis with modified compounds. 

## 4. Materials and Methods

### 4.1. General Procedures

Commercial substrates, namely *p*NP-GlcNAc and *p*NP-GalNAc (Goldbio, St. Louis, CA, USA), GlcNAc (Acros Organics, Geel, Belgium), GalNAc (Glycon Biochemicals, Luckenwalde, Germany), d-glucose (Lach-ner, Neratovice, CZ), d-galactose, *N*-acetylmuramic acid, and d-glucuronic acid (all from Sigma-Aldrich, Munich, Germany), were employed without further purification. If not stated otherwise, other material was from Sigma-Aldrich, Munich, Germany. Thin layer chromatography (TLC) was performed on aluminium sheets precoated with Silica Gel 60 (F254 Merck, D), using 2-propanol/H_2_O/NH_4_OH aq. (7:2:1) as an eluent. Plates were visualized under UV light (254 nm) to detect the presence of UV active compounds and were then charred with a solution of 5% H_2_SO_4_ in ethanol.

### 4.2. Structural Analysis of Compounds

#### 4.2.1. ESI-MS Analysis

Mass spectra were obtained using the Shimadzu Prominence system comprising a Shimadzu CBM-20A system controller, a Shimadzu LC-20AD binary HPLC pump, a Shimadzu CTO-10AS column oven, a Shimadzu SIL-20ACHT cooling autosampler, and a Shimadzu SPD-20MA diode array detector (Shimadzu, Tokyo, Japan). The ESI-MS parameters were as follows: positive and negative mode; ESI interface voltage 4.5 kV; detector voltage 1.15 kV; nebulizing gas flow 1.5 mL·min^−1^; drying gas flow 15 mL·min^−1^; heat block temperature 200 °C; temperature of desolvation line pipe 250 °C; SCAN mode 300–700 *m/z*. The mobile phase (acetonitrile) flow rate was 0.3 mL min^−1^; chromatograms were analyzed using LabSolutions software ver. 5.75 SP2 (Shimadzu, Kyoto, Japan).

#### 4.2.2. NMR Analysis

NMR spectra were acquired using a Bruker Avance III 600 MHz spectrometer (compounds **8**, **9**, and **10**, Bruker, Billica, MA, USA) or a Bruker Avance III 700 MHz spectrometer (compounds **7** and **16**) in D_2_O (99.96 atom % D, VWR Chemicals, Leuven, Belgium) at 30 °C. The residual signal of water (*δ*_H_ 4.508) served as a reference in proton spectra; carbon spectra were referenced to the signal of acetone (*δ*_C_ 30.50). The structural assignment was based on information obtained from COSY, HSQC, HSQC-TOSCY, 1d-TOCSY, and HMBC experiments. The set of extracted vicinal ^3^*J*_H,H_ coupling constants in individual sugar units allowed us to determine their anomeric configuration and to distinguish galactose and glucose moieties. The position of the glycosidic linkage was proved by HMBC contacts between involved carbons and anomeric protons of subsequent sugar units.

### 4.3. Enzymatic Activity Assay

β-*N*-Acetylhexosaminidase activity was determined spectrophotometrically (λ = 420 nm) in an end-point assay, by quantifying the release of 4-nitrophenol in the hydrolysis of *p*NP-GlcNAc and *p*NP-GalNAc (2 mM starting concentration) in McIlvaine (50 mM citrate-phosphate) buffer pH 5. Blank reactions were prepared analogously without an enzyme. Samples were incubated at 35 °C and 850 rpm for 10 min. The reaction must be stopped in time to afford only a low conversion of substrate (≤ ca 10%), in order to ensure the linear dependence of increasing *p*-nitrophenol formation on time. Then, the reactions were stopped by adding 1 mL of 0.1 M Na_2_CO_3_ and the amount of released 4-nitrophenol was determined colorimetrically. One unit of enzyme activity is defined as the amount of enzyme that cleaves 1 μmol of substrate per minute under the assay conditions.

### 4.4. Analytical Transglycosylation Reactions

The screening of transglycosylation reactions with the recombinant wild-type β-*N*-acetylhexosaminidase from *Talaromyces flavus* (*Tf*Hex WT) at an analytical scale (200 μL) was performed in McIlvaine buffer pH 5 in the presence of 50 mM donor and 300 mM acceptor (GlcNAc (**1**), Glc (**2**), MurNAc (**3**), GlcA (**4**), GalNAc (**5**), and Gal (**6**)). The glycosyl donor was *p*NP-GlcNAc, except when the GalNAc acted as the acceptor. *Tf*Hex WT was heterologously produced in *Pichia pastoris* and purified as described previously [13]. The final concentration of enzyme in the reaction mixture was 0.25 U mL^−1^. The reaction mixtures were incubated at 35 °C under shaking at 1000 rpm. The reaction progress was monitored by TLC every 2 h up to 24 h or until all the donor had been consumed. 

Analytical transglycosylation reactions with the *Tf*Hex Y470H mutant enzyme, where Tyr470 was substituted by histidine to suppress hydrolytic activity and promote transglycosylation, were performed similarly. The mutant enzyme was produced and purified as described previously [13]. The *p*NP-GalNAc donor (50 mM) and selected acceptor (GlcA, MurNAc or their glycosides) (100 mM) were suspended in 50 mM McIlvaine buffer pH 5 (final volume 200 μL). The reaction was started by the addition of *Tf*Hex Y470H (0.5 U mL^−1^ final volume) and it was incubated at 35 °C and 1000 rpm.

### 4.5. Preparative Transglycosylation Reactions

#### 4.5.1. 2-Acetamido-2-Deoxy-β-d-Glucopyranosyl-(1-4)-2-Acetamido-2-Deoxy-d-Glucopyranose (GlcNAc-β(1-4)-GlcNAc; **7**)

*p*NP-GlcNAc (3 × 17 mg, 50 mM) and the GlcNAc (**1**) acceptor (3 × 66 mg, 300 mM) were suspended in 3 × 1 mL of McIlvaine buffer pH 5 and the mixture was incubated at 35 °C and 1000 rpm. The reaction was started by adding *Tf*Hex WT (3 × 0.25 U; activity related to hydrolysis of *p*NP-GlcNAc). Reactions were monitored by TLC (Figure S1, Supplementary Materials) and stopped after 5 h by heating at 99 °C for 3 min. Reaction mixtures were cooled down to room temperature and centrifuged at 13,500 rpm for 10 min; the supernatant was loaded onto a Biogel-P2 column (15 × 900 mm) with water as a mobile phase. Upon analysis by TLC, fractions containing product **7** were combined and the remaining traces of *p*-nitrophenol were removed by extraction into ethyl acetate. After lyophilization, disaccharide 7 was obtained as a white solid (15 mg, 24% yield). ESI-MS: found *m*/*z* 521 for [M + HSO_4_]^−^, calcd for C_16_H_29_N_2_O_15_S 521.1. ^1^H and ^13^C NMR data were consistent with the structure and were compared to the literature [19].

#### 4.5.2. 2-Acetamido-2-Deoxy-β-d-Galactopyranosyl-(1-6)-2-Acetamido-2-Deoxy-d-Galactopyranose (GalNAc-β(1-6)-GalNAc; **8**)

*p*NP-GalNAc (3 × 17 mg, 50 mM) and the GalNAc (**5**) acceptor (3 × 66 mg, 300 mM) were suspended in 3 × 1 mL of McIlvaine buffer pH 5 and the mixture was incubated at 35 °C at 1000 rpm. The reaction was started by the addition of *Tf*Hex WT (3 × 0.25 U; activity related to hydrolysis of *p*NP-GalNAc). Reactions were monitored by TLC (Figure S2, Supplementary Materials) and stopped after 5.5 h by heating at 99 °C for 3 min. Reaction mixtures were cooled down to room temperature and centrifuged at 13,500 rpm for 10 min. Then, the supernatant was loaded onto a Biogel-P2 column (15 × 900 mm) with water as a mobile phase. Upon analysis by TLC, fractions containing product **8** were pooled together and the remaining traces of *p*-nitrophenol were removed by extraction into ethyl acetate. Lyophilization afforded product **8** as a white solid (19 mg, 31% yield). ESI-MS: found *m*/*z* 447 for [M + Na]^+^, calcd for C_16_H_28_N_2_NaO_11_ 447.2; found *m*/*z* 521 for [M + HSO_4_]^−^, calcd for C_16_H_29_N_2_O_15_S 521.1. The ^1^H and ^13^C NMR results are shown in the Supplementary Materials (Table S1, Figure S14a,b).

#### 4.5.3. 2-Acetamido-2-Deoxy-β-d-Glucopyranosyl-(1-1)-β-d-Glucopyranoside (GlcNAc-β(1-1)-Glc; **9**)

The *p*NP-GlcNAc donor (3 × 17 mg, 50 mM) and Glc (**2**) acceptor (3 × 54 mg, 300 mM) were suspended in 3 × 1 mL of McIlvaine buffer pH 5 and the mixture was incubated at 35 °C at 1000 rpm. The reaction was started by the addition of *Tf*Hex WT (3 × 0.25 U; activity related to hydrolysis of *p*NP-GlcNAc). Reactions were monitored by TLC (Figure S3, Supplementary Materials) and stopped after 5.5 h when the donor was almost consumed by heating at 99 °C for 3 min. Reaction mixtures were cooled down to room temperature and centrifuged for 10 min at 13,500 rpm to remove the denatured enzyme. Then, the supernatant was loaded onto a Biogel-P2 column (15 × 900 mm) with water as a mobile phase. On the basis of TLC analysis, fractions containing product **9** were combined and extracted with ethyl acetate to remove traces of *p*-nitrophenol until a colorless solution was obtained. Since the lyophilized product fraction contained unwanted *p*-nitrophenyl *N*,*N*′-diacetylchitobioside (autocondensation product of *p*NP-GlcNAc) according to TLC, the second purification step was applied using an Amberlite XAD-2 (Sigma-Aldrich, Munich, Germany) column, eluted with water. The eluted fractions, void of aromatic impurities, were collected and lyophilized, to obtain impure non-reducing disaccharide **9** as a white solid (8 mg, approximate yield 14%). The sample of compound **9** contained other by-products, probably regioisomers, which could not be identified. ESI-MS: found *m*/*z* 384 for [M + H]^+^, calcd for C_14_H_26_NO_11_ 384.1; found *m*/*z* 406 for [M + Na]^+^, calcd. 406.1 for C_14_H_25_NNaO_11_; found *m/z* 418 for [M + Cl]^−^, calcd. 418.1 for C_14_H_25_ClNO_11_. ^1^H and ^13^C NMR data are shown in the Supplementary Materials (Table S2, Figure S15a,b).

#### 4.5.4. 2-Acetamido-2-Deoxy-β-d-Glucopyranosyl-(1-1)-β-d-Galactopyranoside (GlcNAc-β(1-1)-Gal; **10**)

The preparative reaction with the Gal (**6**) acceptor (Figure S4, Supplementary Materials) was performed analogously to the Glc acceptor, only using Gal (3 × 54 mg, 300 mM). Purification was performed analogously to compound **9** and it afforded compound **10** as a white solid (6 mg, 10% yield). The sample of compound **10** contained other by-products, probably regioisomers, which could not be identified. ESI-MS: found *m*/*z* 384 for [M + H]^+^, calcd for C_14_H_26_NO_11_ 384.1; found *m*/*z* 406 for [M + Na]^+^, calcd. 406.1 for C_14_H_25_NNaO_11_; found *m/z* 418 for [M + Cl]^−^, calcd. 418.1 for C_14_H_25_ClNO_11_. The ^1^H and ^13^C NMR results are shown in the Supplementary Materials (Table S3, Figure S16a,b).

#### 4.5.5. 2-Acetamido-2-Deoxy-β-d-Glucopyranosyl *N*-Acetylmuramic acid (GlcNAc-β(1-X)-MurNAc)

The *p*NP-GlcNAc donor (3 × 14 mg, 50 mM) and MurNAc (**3**) acceptor (3 × 24 mg, 100 mM) were suspended in 3 × 0.8 mL of McIlvaine buffer pH 5. After dissolving MurNAc, the pH of the reaction mixture was re-adjusted to pH 5 and the mixture was incubated at 35 °C at 1000 rpm. The reaction was started by the addition of *Tf*Hex Y470H (3 × 1.3 U; activity related to hydrolysis of *p*NP-GlcNAc). Reactions were monitored by TLC (Figure S5, Supplementary Materials). After 3.5 h, another portion of donor was added (3 × 14 mg) and stopped after 7 h when the donor was almost consumed by heating at 99 °C for 3 min. Reaction mixtures were cooled down to room temperature and centrifuged for 10 min at 13,500 rpm to remove the denatured enzyme. Then, the supernatant was loaded onto a Biogel-P2 column (15 × 900 mm) with water as a mobile phase. TLC analysis showed that the desired product migrated together with the MurNAc acceptor. Fractions containing the product were combined and extracted with ethyl acetate to remove traces of *p*-nitrophenol. Lyophilization afforded partially purified product, as identified by ESI-MS analysis. ESI-MS: found *m*/*z* 497 for [M + H]^+^, calcd for C_19_H_33_N_2_O_13_ 497.2; found *m*/*z* 495 for [M − H]^-^, calcd for C_19_H_31_N_2_O_13_ 495.2. Unfortunately, due to a high concentration of the MurNAc acceptor in the partially purified product, NMR analysis could not be performed.

#### 4.5.6. Propyl 2-Acetamido-2-Deoxy-β-d-Glucopyranosyl-(1-6)-*N*-Acetylmuramic acid (GalNAc-β(1-6)-MurNAc-*O*Pr; **16**)

*p*NP-GalNAc (3 × 17 mg, 50 mM) and the MurNAc-*O*Pr (**13b**) acceptor (3 × 34 mg, 100 mM) were suspended in 3 × 1 mL McIlvaine buffer pH 5 and the mixture was incubated at 35 °C at 1000 rpm. The reaction was started by the addition of *Tf*Hex Y470H (3 × 0.5 U, related to hydrolysis of *p*NP-GalNAc activity). Reactions were monitored by TLC (Figure S6, Supplementary Materials) and stopped after 3 h by heating at 99 °C for 3 min. Then, reaction mixtures were cooled down to room temperature and centrifuged at 13,500 rpm for 10 min. Following this, the supernatant was loaded onto a Biogel-P2 column (15 × 900 mm) with water as a mobile phase. Upon TLC analysis, fractions containing product **16** were combined and remaining traces of *p*-nitrophenol were extracted into ethyl acetate. After lyophilization, a second purification step, flash chromatography on silica gel, was conducted, using chloroform/methanol/NH_4_OH_(aq)_ in a ratio of 2:3:0.4 as mobile phase. Fractions were analyzed by TLC and those containing product **16** were pooled together and evaporated in vacuo to yield disaccharide **16** (2 mg, 1% yield). ESI-MS: found *m*/*z* 557 for [M – H + H_2_O]^+^, calcd for C_22_H_41_N_2_O_14_ 557.2. The ^1^H and ^13^C NMR results are shown in the Supplementary Materials (Table S4, Figure S17a,b).

### 4.6. Docking and Molecular Dynamics of the TfHex Active Site in the Complex with Tested Acceptors

The models used herein were adopted from those previously published [20]. The orientation of long loops and the *N*-terminal domain were corrected based on the crystal from *Aspergillus oryzae*; pdb: 5oar [17]. Molecular dynamics simulations were done for WT and Y470H mutants with docked GlcNAcox and GalNAcox ligands in the active site. Both oxazolines were docked into each monomer of the dimeric structure of *Tf*Hex and analyzed for 10 ns of molecular dynamics simulation, with the parameters explained in [13]. Several models with docked oxazolines were selected from the stable period of simulation (Figure S8, Supplementary Materials) for docking aglycones (each monomer was analyzed separately). To extend sampling of aglycone binding place, we analyzed several molecular dynamics snapshots with various Glu371, Trp509, and transglycosylation donor orientations, which could influence aglycone binding [21]. We defined two main parameters for the selection of enzyme–donor complexes from a production molecular dynamics run: distance from the catalytic Glu371 carboxy group and C-1 of oxazoline (Figure S9, Supplementary Materials), and the dihedral angle formed by CA-CB-CG-OE2 atoms in Glu 371. Structures with various dihedral angles and various distances were chosen, minimized with Yasara [22], and used for further docking experiments.

We performed the constraint and free docking with Glide [23]. Constraints for docking were as follows: distance between Glu371 (OE2 atom) and hydroxyl of the transglycosylation acceptor, and distance from hydroxyl of the acceptor to C-1 of the donor. The selection of these parameters was based on the analysis of the second step of hydrolysis/transglycosylation. During this step, two main events occur: donation of hydrogen to Glu371 from the acceptor/water with subsequent attack at C-1 of the donor [24]. An increase of these distances complicates the attack at donor C-1 and disallows the formation of the transglycosylation product [21]. Substrates were bound with a local docking algorithm with a grid, centered at Glu371 and Trp509, and extended in all directions by 10 Å from the center. Docked poses were scored with an SP Glide score and at least the five best structures were reported for each run. Docked poses with a proper geometry for transglycosylation and with the best binding scores were selected. Scores for structures with different Glu371 orientations were scaled based on the probability of occurrence, according to Equation (1):

(1) $$\mathrm{Scaled}\;\mathrm{score}=P/n\sum_{i=1}^{n}SP_{Glide\;score},$$

where *P* is the probability of observing a certain rotameric conformation of catalytic Glu371 during the production run, and *n* is the number of different orientations of catalytic residues. The change of Glu371 orientation with respect to the docked intermediate reflects the influence of the docking orientation of the transglycosylation acceptor and the docking score. To cover the possible catalytic residue conformations during molecular dynamics, we selected several representative structures with different Glu371 rotamers (Figure S9). For the molecular dynamics simulations with WT-GalNAcox and WT-GlcNAcox, the root mean square deviation of the compound was already stabilized after 3.5 ns; the period of 3.5–10 ns was used for calculation probabilities.

If a docking pose is found in all analyzed conformations, its score is below −4 kcal/mol. In case the mentioned orientation is unavailable in some conformations, the score is increased. As a result, for a good docking pose found in 50% MD of simulation snapshots, the scaled scores would be lower than −2 kcal/mol. This criterion was used for estimating a significant value to be reported.

The binding pocket surface was calculated by the Site map for the region within 3 Å, and extended to the distance of 3 Å to the solvent.

Refinement of the docked positions of transglycosylation acceptors was done by a molecular dynamics simulation (10–15 ns) with Yasara [22]. One repetition was conducted for each distinct orientation, with molecular dynamics parameters used for oxazolines.

## 5. Conclusions

The present work provides a rational explanation for the transglycosylation acceptor specificity of the *N*-acetylhexosaminidase from *Talaromyces flavus*. First, we found that the *N*-acetamido group is a crucial structural feature in the acceptor molecule. Both GlcNAc and GalNAc acceptors are selectively glycosylated with good yields. With the GalNAc acceptor, the enzyme exhibits a shift of its classic β(1-4) regioselectivity to β(1-6), which accords with the hypothesis that β-*N*-acetylhexosaminidases are unable to glycosylate the axial C-4 hydroxyl. In contrast, the respective structural counterparts Glc and Gal lacking the *N*-acetamido group yield a complex product mixture containing unique non-reducing disaccharides GlcNAc-β(1-1)-Glc or GlcNAc-β(1-1)-Gal, respectively, among the major products. Furthermore, it was found that tMurNAc, GlcA, and their β-glycosides are hardly or not tolerated as glycosylation acceptors by the WT enzyme. Fortunately, the Y470H mutant enzyme was able to recognize the MurNAc-*O*Pr acceptor, affording the respective β(1-6) linked disaccharide with a low yield. Molecular modeling studies indicate that a long substituent at C-1, such as propyl, is beneficial for the acceptor specificity of *Tf*Hex. The GalNAc-β(1-6)-MurNAc-*O*Pr disaccharide is the first enzymatically glycosylated MurNAc derivative and it opens new possibilities in enzymatic synthesis with modified MurNAc derivatives.

## Figures and Tables

**Table 1:** Preparative transglycosylation reactions with *Tf*Hex WT and Y470H.

Entry	Enzyme	Donor	Acceptor	Donor/Acceptor [mM] ^1^	Product	Yield
**I**	WT	*p*NP-GlcNAc	GlcNAc	50/300	GlcNAc-β(1-4)-GlcNAc	24%
**II**	WT	*p*NP-GalNAc	GalNAc	50/300	GalNAc- β (1-6)-GalNAc	31%
**III**	WT	*p*NP-GlcNAc	Glc	50/300	GlcNAc- β (1-1)-Glc	14%
**IV**	WT	*p*NP-GlcNAc	Gal	50/300	GlcNAc- β (1-1)-Gal	10%
**V**	Y470H	*p*NP-GlcNAc	MurNAc	50 (x2)/100	GlcNAc- β (1-X)-MurNAc	n.q. ^1^
**VI**	Y470H	*p*NP-GalNAc	MurNAc-*O*Pr	50/100	GalNAc- β (1-6)-MurNAc-*O*Pr	≈1%

^1^ Not quantified. The product could not be isolated in a sufficient purity for NMR characterization.

**Table 2:** Scaled binding scores of docked transglycosylation acceptors (kcal/mol). The orientation of site 1 and site 2 is described in the text.

Acceptor	Position of Respective Acceptor Hydroxyl close to Catalytic Glu371						
	None (Site 1)	None (Site 2)	C-1	C-2	C-3	C-4	C-6
***Tf*Hex WT in complex with GlcNAcox/GalNAcox**							
GlcNAc (**1**)	n.b.^1^/**−3.99**	−2.28/n.b.	n.b./n.b.	n.b./n.b.	−2.37/n.b.	**−4.97/**−3.36	−2.29/−2.09
Glc (**2**)	**−5.24/−2.68**	n.b./n.b.	-2.35/n.b.	-2.14/n.b.	−4.44/n.b.	−4.70/n.b.	−2.27/n.b.
MurNAc (**3**)	n.b./** −4.46**	**−4.75/**−2.93	n.b./n.b.	n.b./n.b.	n.b./n.b.	n.b./n.b.	−3.66/n.b.
GlcA (**4**)	**−5.25/−5.47**	−2.31/−2.60	n.b./n.b.	-2.03/n.b.	n.b./n.b.	−2.221/n.b.	n.b./n.b.
GalNAc (**5**)	**−4.98/−2.62**	−2.65/n.b.	n.b./n.b.	n.b./n.b.	−4.03/−2.28	n.b./n.b.	n.b./−2.23
Gal (**6**)	−2.57/**−3.47**	−2.36/n.b.	-2.29/n.b.	n.b./n.b.	**−2.81**/n.b.	n.b./n.b.	n.b./n.b.
MurNAc-*O*Pr (**XX**)	−2.42/**−4.34**	**−4.41/**−2.66	n.b./n.b.	n.b./n.b.	n.b./n.b.	n.b./n.b.	−4.12/−2.93
***Tf*Hex Y470H in complex with GlcNAcox/GalNAcox**							
GlcA (**4**)	−5.45/3.80	n.b./**−5.36**	n.b./n.b.	n.b./n.b.	n.b./n.b.	n.b./n.b.	n.b./n.b.
MurNAc (**3**)	n.b./n.b.	**−4.68**/**−4.61**	n.b./n.b.	n.b./n.b.	n.b./n.b.	n.b./n.b.	n.b./−2.69
MurNAc-*O*Pr (**XX**)	−2.96/−3.19	**−5.05**/**−4.50**	n.b./n.b.	n.b./n.b.	n.b./n.b.	n.b./n.b.	n.b./−3.69

^1^ n.b. stands for not bound. Two most favorable binding scores for each complex are indicated in red; the scores corresponding to bonds confirmed by the synthetic experiment are underlined.

## Supplementary Materials

Supplementary Materials can be found at https://www.mdpi.com/1422-0067/20/24/6181/s1.

## Abbreviations

ESI-MS	Electrospray ionization mass spectrometry
Gal	Galactose
GalNAc	*N*-Acetylgalactosamine
GalNAcox	*N*-Acetylgalactosamine oxazoline (catalytic intermediate during enzymatic hydrolysis)
Glc	Glucose
GlcA	Glucuronic acid
GlcNAc	*N*-Acetylglucosamine
GlcNAcox	*N*-Acetylglucosamine oxazoline (catalytic intermediate during enzymatic hydrolysis)
HB	Hydrogen bond
MurNAc	*N*-acetylmuramic acid
MurNAc-*O*Pr	Propyl glycoside of *N*-acetylmuramic acid
*p*NP-GalNAc	4-Nitrophenyl *N*-acetyl-β-d-galactosaminide
*p*NP-GlcNAc	4-Nitrophenyl *N*-acetyl-β-d-glucosaminide
*Tf*Hex WT	Wild type β-*N*-acetylhexosaminidase from *Talaromyces flavus*
*Tf*Hex Y470H	Mutant β-*N*-acetylhexosaminidase from *Talaromyces flavus*, Tyr470 is exchanged for His
