# Supplementary material for: Acceptor Specificity of β-N-Acetylhexosaminidase from Talaromyces flavus: A Rational Explanation
Source: Int J Mol Sci. 2019 Dec 7;20(24):6181. doi: 10.3390/ijms20246181 (PMC6940953; doi:10.3390/ijms20246181)
Supplement: Supplementary file 1 [file ijms-20-06181-s001.pdf]

International Journal of
Molecular Sciences

MDPI

Supplementary Materials

Acceptor specificity of β-*N*-acetylhexosaminidase from *Talaromyces flavus*: a rational explanation

Cecilia Garcia-Oliva[1], Pilar Hoyos[1], Lucie Petrásková[2], Natalia Kulik[3], Helena Pelantová[2], Alfredo H. Cabanillas[4], Ángel Rumbero[4], Vladimir Křen [2] María J. Hernáiz[1]* and Pavla Bojarová[2]*

[1] Department of Chemistry in Pharmaceutical Sciences, Faculty of Pharmacy, Complutense University of Madrid, Plaza Ramón y Cajal, E 28040 Madrid, Spain; mjhernai@ucm.es

[2] Institute of Microbiology of the Czech Academy of Sciences, Vídeňská 1083, CZ 14220 Prague 4, Czech Republic; bojarova@biomed.cas.cz

[3] Center for Nanobiology and Structural Biology, Institute of Microbiology, Czech Academy of Sciences, Zámek 136, CZ 37333 Nové Hrady, Czech Republic; kulik@nh.cas.cz

[4] Department of Organic Chemistry, Autonomous University of Madrid, Cantoblanco, 28049 Madrid, Spain; angel.rumbero@uam.es

***** Correspondence: bojarova@biomed.cas.cz; Tel: +420-296-442-360 (P.B.); mjhernai@ucm.es; tel.: +34-9139-418-20 (M.J.H.)

Received: date; Accepted: date; Published: date

Abstract: Fungal β-*N*-acetylhexosaminidases, though hydrolytic enzymes *in vivo*, are useful tools in the preparation of oligosaccharides of biological interest. The β-*N*-acetylhexosaminidase from *Talaromyces flavus* is remarkable for its synthetic potential, broad substrate specificity and tolerance to substrate modifications. It can be heterogously produced in *Pichia pastoris* in a high yield. The mutation of Tyr470 residue to histidine greatly enhances its transglycosylation capability. The aim of this work is to identify the structural requirements of this model β-*N*-acetylhexosaminidase for its transglycosylation acceptors, and formulate a structure-activity relationship study. Enzymatic reactions were performed using an activated glycosyl donor, 4-nitrophenyl *N*-acetyl-β-D-glucosaminide or 4-nitrophenyl *N*-acetyl-β-D-galactosaminide, and a panel of glycosyl acceptors of varying structural features (*N*-acetylglucosamine, glucose, *N*-acetylgalactosamine, galactose, *N*-acetylmuramic acid, and glucuronic acid). The transglycosylation products were isolated and structurally characterized. The C-2 *N*-acetamido group in the acceptor molecule was found to be essential for recognition by the enzyme. The presence of C-2 hydroxyl moiety strongly hindered the normal course of transglycosylation, yielding unique non-reducing disaccharides in a low yield. Moreover, whereas the *gluco*-configuration at C-4 steered the glycosylation into β(1-4) position, the *galacto*-acceptor afforded β(1-6) glycosidic linkage. The Y470H mutant enzyme was tested with acceptors based on β-glycosides of uronic acid and *N*-acetylmuramic acid. With the latter acceptor, were able to isolate and characterize one glycosylation product with a low yield. To our knowledge, this is the first example of an enzymatic glycosylation of an *N*-acetylmuramic acid derivative. In order to explain these findings and predict the enzyme behavior, a modeling study was accomplished that correlated with the acquired experimental data.

Contents:

1. Transglycosylation reaction mechanism of the β-*N*-acetylhexosaminidase from *Talaromyces flavus*
2. Progress of transglycosylation reactions monitored by thin layer chromatography
3. Docking and molecular dynamics simulations
4. Structural characterization of compounds
5. Abbreviations

1. Transglycosylation reaction mechanism of the β-*N*-acetylhexosaminidase from *Talaromyces flavus*

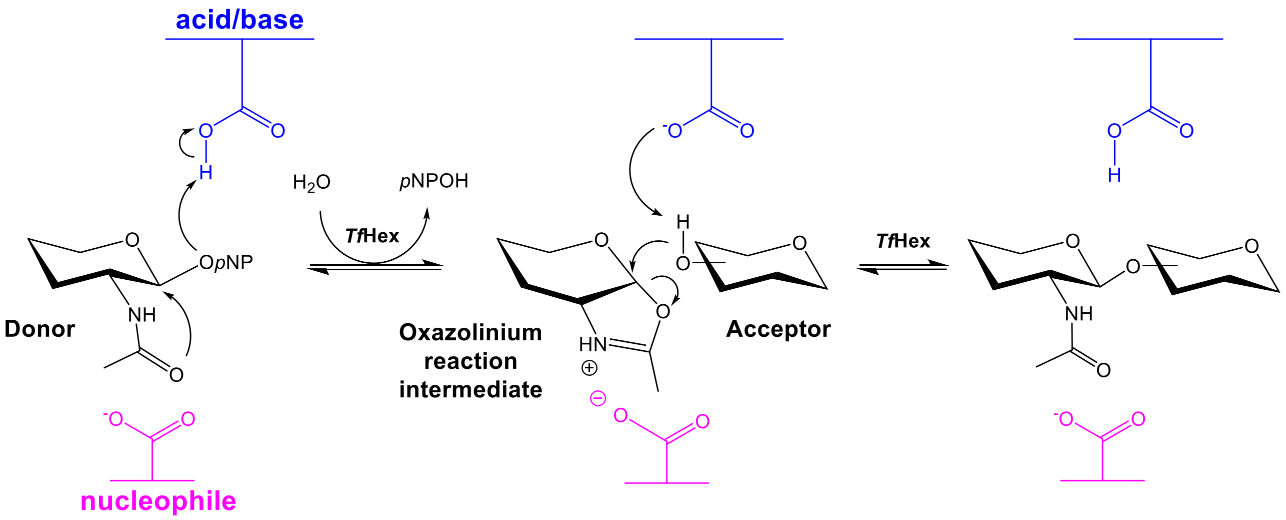

Scheme S1. Substrate-assisted mechanism of transglycosylation utilized by *Tf*Hex. The two key catalytic residues are Asp370 (nucleophile), and Glu371 (acid/base). The catalytic nucleophile does not directly participate in the catalysis as in the case of classical retaining glycosidases but rather stabilizes the oxazoline reaction intermediate.

2. Progress of transglycosylation reactions monitored by thin layer chromatography

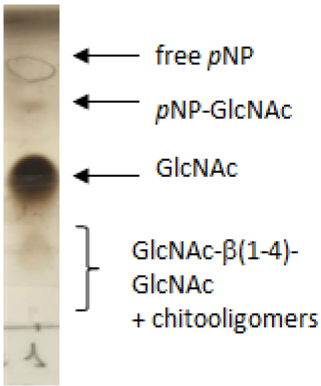

Figure S1. Reaction of *p*NP-GlcNAc (3×17 mg, 50 mM) with GlcNAc (**1**; 3×66 mg, 300 mM) catalyzed by *Tf*Hex WT (3x0.25 U), affording product **7** (GlcNAc-β(1-4)-GlcNAc). Reaction time: 5 h. Reaction volume: 3x1 mL.

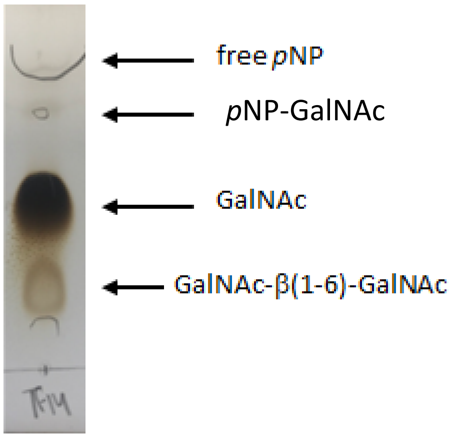

Figure S2. Reaction of *p*NP-GalNAc (3x17 mg, 50 mM) with GalNAc (**5**; 3x66 mg, 300 mM) catalyzed by *Tf*Hex WT (3x0.25 U), affording product **8** (GalNAc-β(1-6)-GalNAc). Reaction time: 5.5 h. Reaction volume: 3x1 mL.

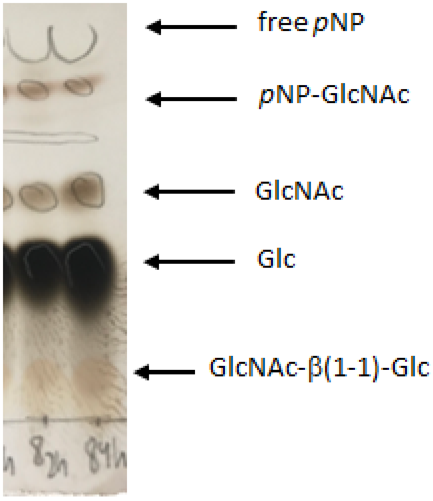

Figure S3. Reaction of *p*NP-GlcNAc (3x 17 mg, 50 mM) with Glc (**2**; 3x54 mg, 300 mM) catalyzed by *Tf*Hex WT (3x0.25 U), affording product **9** (GlcNAc-β(1-1)-Glc). Reaction time: 5.5 h. Reaction volume: 3x1 mL.

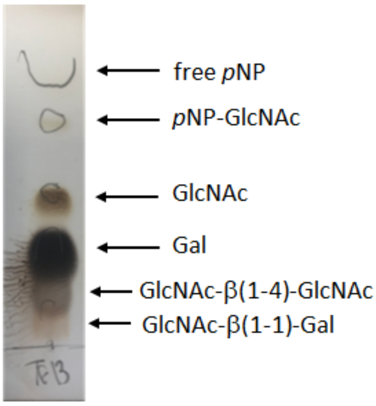

Figure S4. Reaction of *p*NP-GlcNAc (3x17 mg, 50 mM) with Gal (**6**; 3x54 mg, 300 mM) catalyzed by *Tf*Hex WT (3x0.25 U), affording product **10** (GlcNAc-β(1-1)-Gal). Reaction time: 5.5 h. Reaction volume: 3x1 mL.

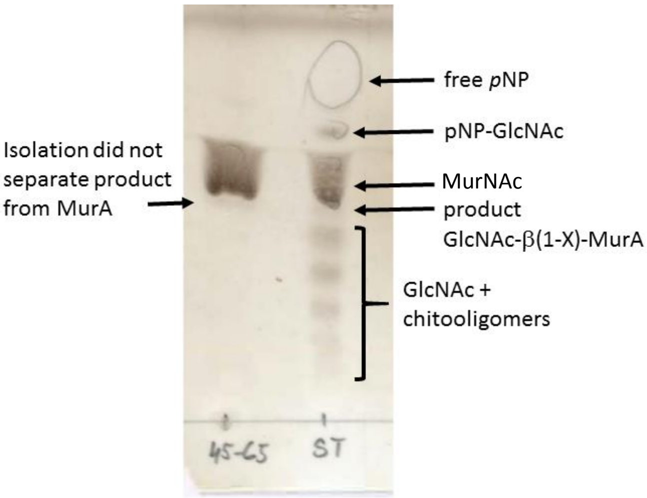

Figure S5. Reaction of *p*NP-GlcNAc (3x14 mg, 50 mM; after 3.5h another batch was added) with MurNAc (**3**; 3x24 mg, 100 mM) catalyzed by *Tf*Hex Y470H (3x1.3 U), affording GlcNAc-β(1-X)-MurNAc. Reaction time: 7 h. Reaction volume: 3x0.8 mL.

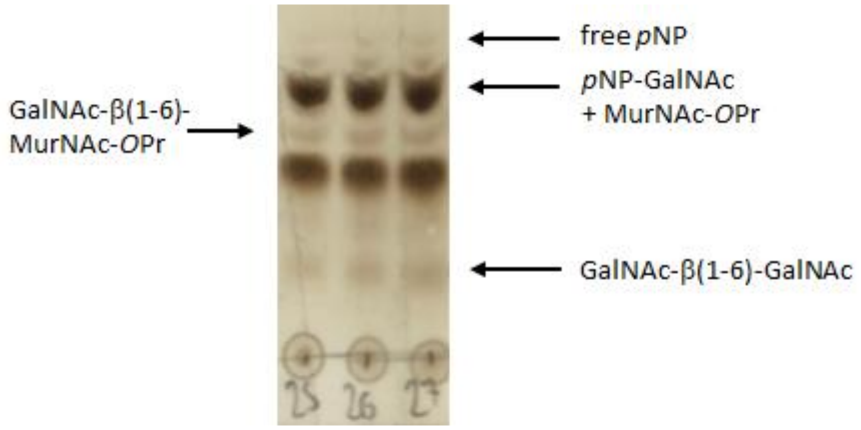

Figure S6. Reaction of *p*NP-GalNAc (3x17 mg, 50 mM) with MurNAc-*O*Pr (**13b**; 3x34 mg, 100 mM) catalyzed by *Tf*Hex Y470H 3x0.5 U), affording product **16** (GalNAc-β(1-6)-MurNAc-*O*Pr). Reaction time: 3 h. Reaction volume: 3x1 mL.

3. Docking and molecular dynamics simulations

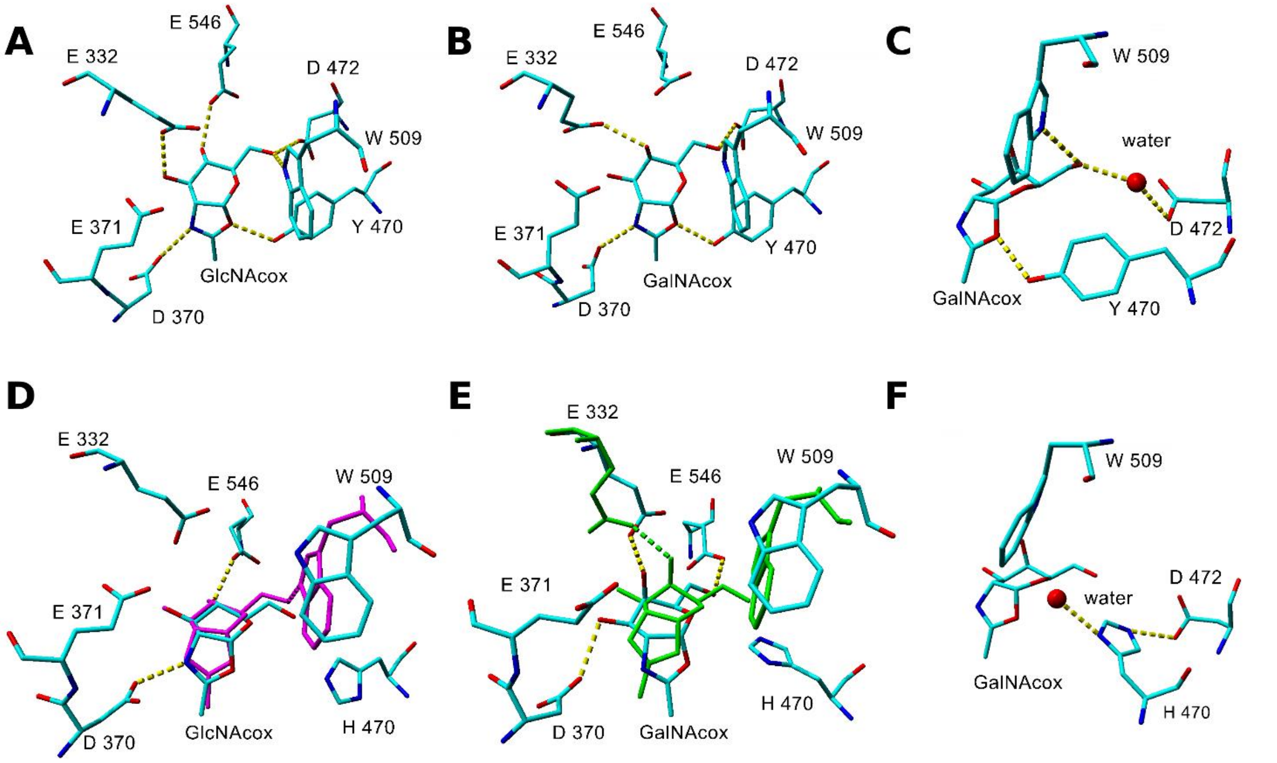

Figure S7. Interactions in the complexes of WT and Tyr470His variants with oxazoline intermediates. Active site amino acids are show as sticks, hydrogen bonds are shown in yellow dashed lines, hydrogens are hidden, and the water molecule is shown in a red ball. (**A**), GlcNAcox in *Tf*Hex WT after 5 ns of molecular dynamics simulation. (**B**), GalNAcox in *Tf*Hex WT after 5 ns of molecular dynamics simulation; the HB with Gln546 with GlcNAcox is lost, and the interaction of Glu332 with GlcNAcox changed. (**C**), Orientation of Tyr470 in the complex of GalNAcox with *Tf*Hex WT after 5 ns of simulation, showing interactions of Tyr470, Trp509, Asp472, and a water molecule; Asp472 forms a direct or a water-mediated interaction with GalNAcox. (**D**), Overlay of the complexes of GlcNAcox with Y470H *Tf*Hex (**element color**) and with *Tf*Hex WT (**magenta**) after 5 ns of simulation, showing residues that changed interaction with oxazoline or their orientation compared to the WT complex. (**E**), Overlay of the complexes of GalNAcox with Y470H *Tf*Hex (**element color**) and with *Tf*Hex WT (**green**) after 5 ns of molecular dynamics simulations, showing residues that changed interaction with oxazoline or their orientation compared to the WT complex. (**F**), Orientation of His470 in the complex of GalNAcox with Y470H *Tf*Hex after 5 ns of simulation, showing interactions of His470, Trp509, Asp472, and a water molecule; His470 is rotated towards Asp472, it lost HB with GalNAcox and attracted a water molecule.

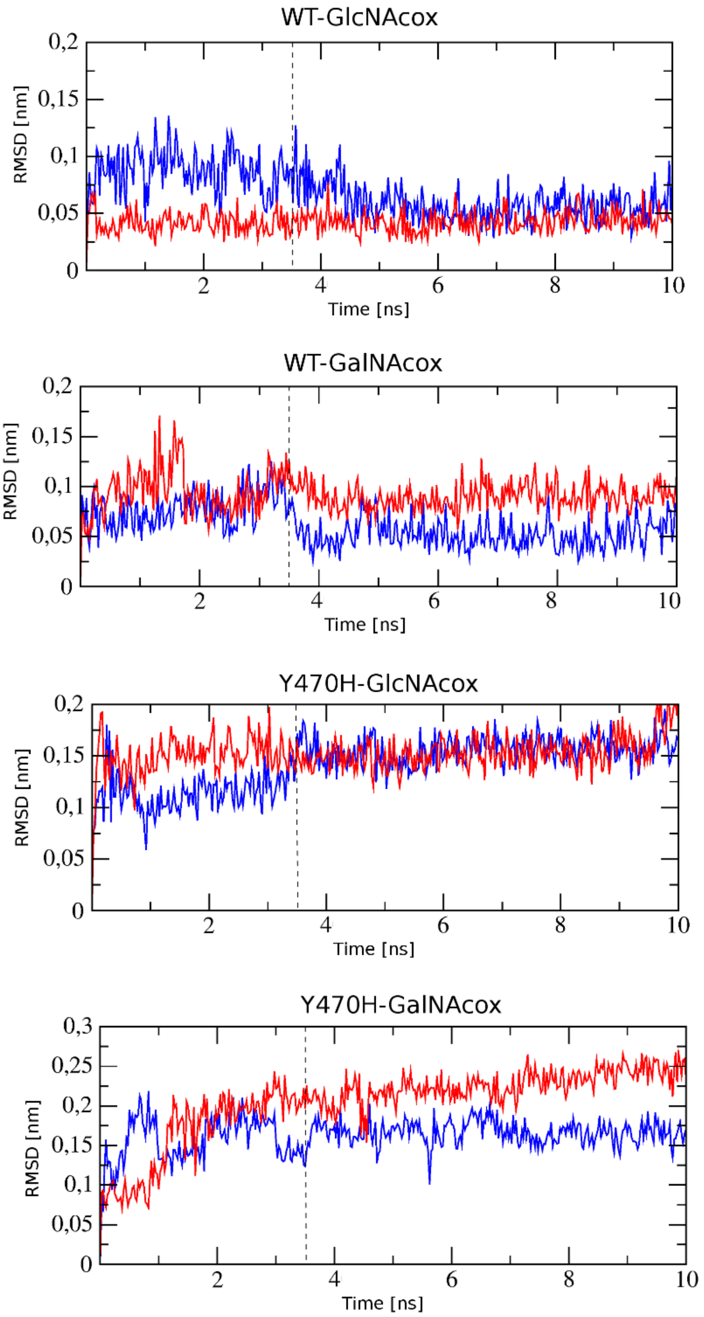

Figure S8. Root means square deviation (RMSD) of intermediate state mimics (GlcNAcox and GalNAcox) during molecular dynamics run. Equilibrated structures correspond to stable RMSD values. The dashed line divides molecular dynamics simulation of the equilibration period (left) and of the analyzed production run (right). The **blue** line corresponds to RMSD of the respective oxazoline docked in monomer 1 and the **red** line corresponds to docking to monomer 2 of the dimeric structure of *Tf*Hex.

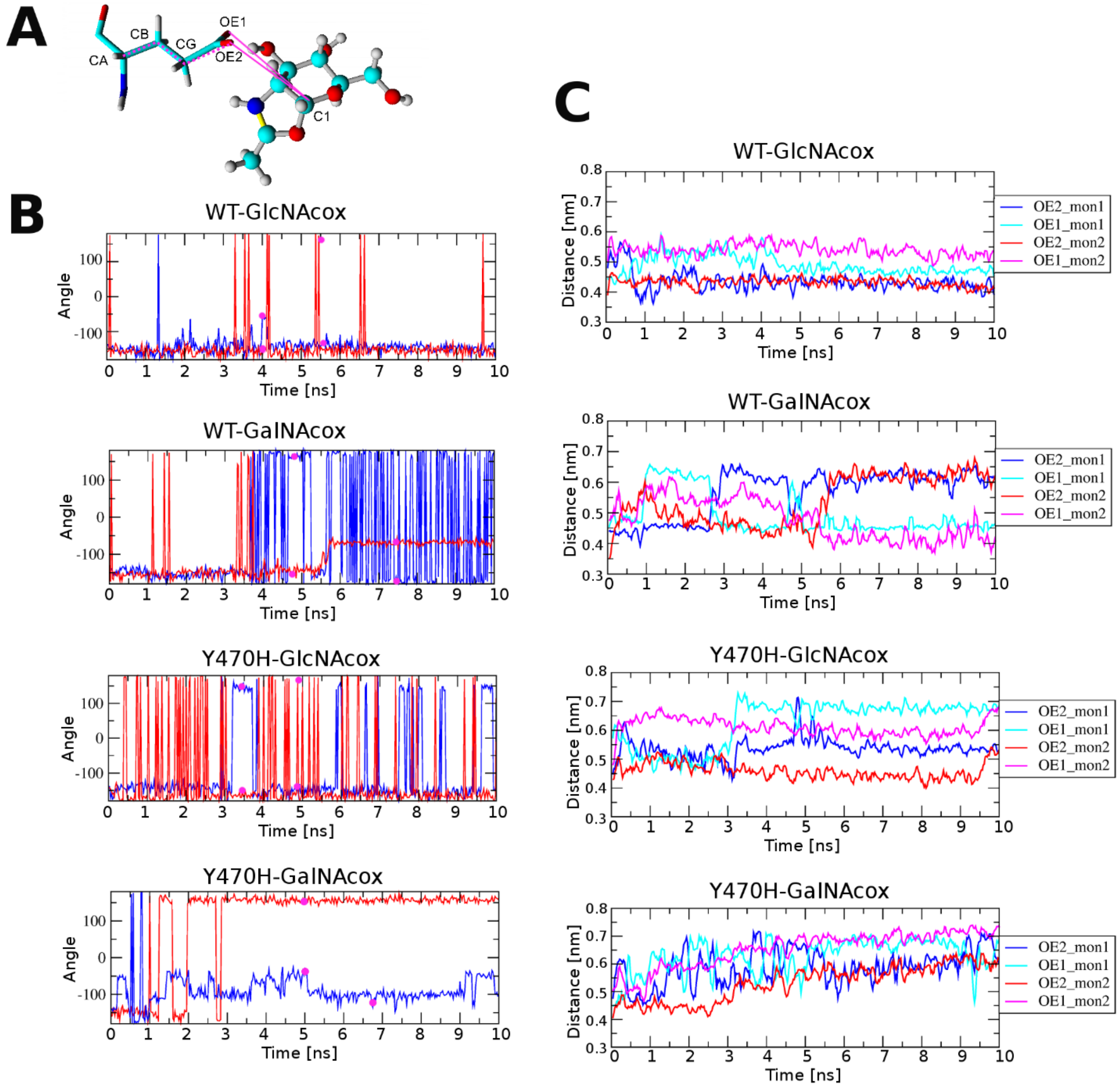

Figure S9. (**A**), Labeling of atoms in catalytic Glu371 and GalNAcox/GlcNAcox used in Yasara. The measured dihedral angle in catalytic Glu371 is shown in magenta dotted lines, respective distances in magenta full lines. (**B**), The dihedral angle in the catalytic residue Glu371 formed by CA-CB-CG-OE2 atoms in the WT and Y470H mutant enzymes complexed with GlcNAc oxazoline and GalNAc oxazoline during MD. **Blue** line corresponds to monomer 1 and **red** line to monomer 2 of the enzyme dimeric structure. Selected representative structures are marked by **magenta** dots. (**C**), Distance from OE1 and OE2 atoms of catalytic Glu371 and C-1 of GlcNAc oxazoline and GalNAc oxazoline.

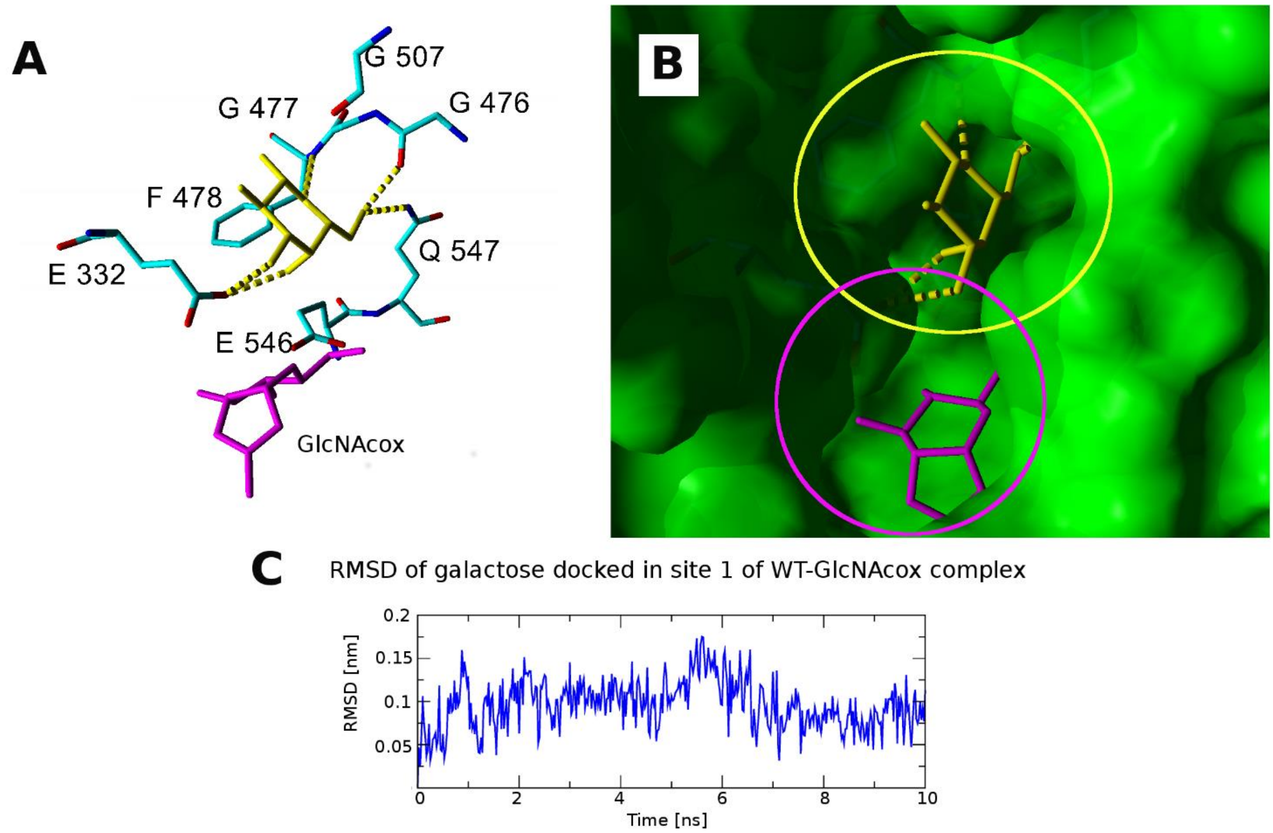

Figure S10. (**A**), Interaction of Gal in the unproductive site 1 in WT-GlcNAcox complex after 10 ns of molecular dynamics. GlcNAc oxazoline is in **magenta**, galactose in yellow. Hydrogen bonds are in yellow dashed lines. **B.** Surface representation of unproductive site 1. Yellow circle shows site 1, **magenta** circle shows the active site. (**C**), RMSD of Gal in site 1 during 10 ns of molecular dynamics simulation.

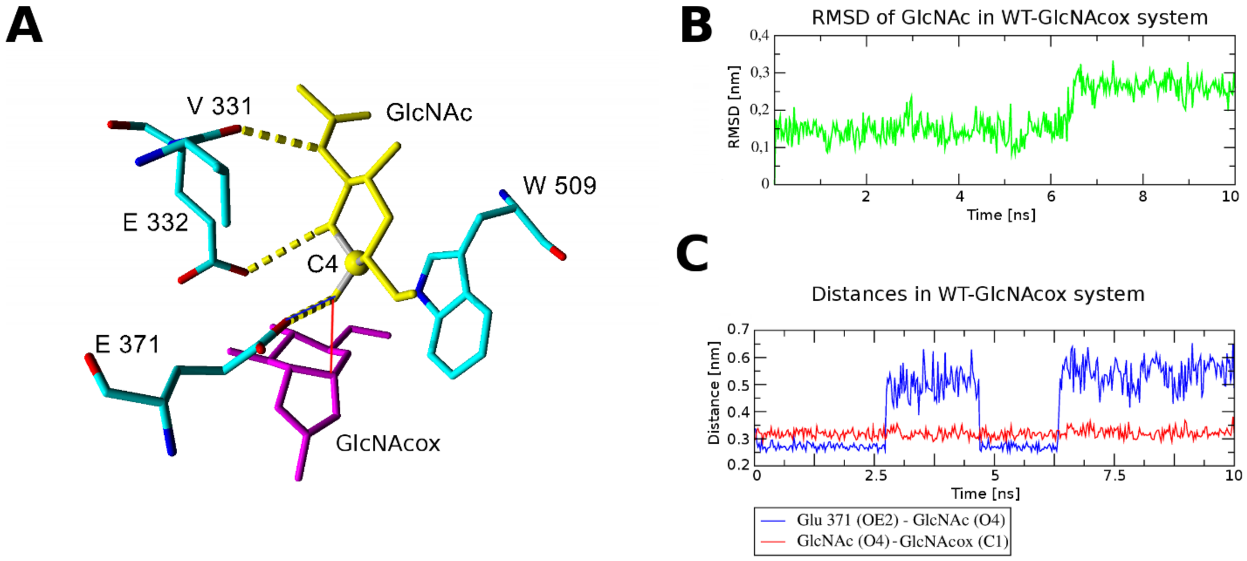

Figure S11. (**A**), Snapshot from molecular dynamics simulation of WT-GlcNAcox complex with GlcNAc acceptor (orientation with C-4 close to Glu 371) after 10 ns. HB are shown in yellow dashed lines. (**B**), RMSD of GlcNAc acceptor in WT-GlcNAcox complex during 10 ns MD. (**D**), Distance between O4 of GlcNAc and OE2 atom of Glu371 (**blue**) or C-1 of GlcNAc oxazoline (**red**) in the WT-GlcNAcox complex with docked GlcNAc during molecular dynamics.

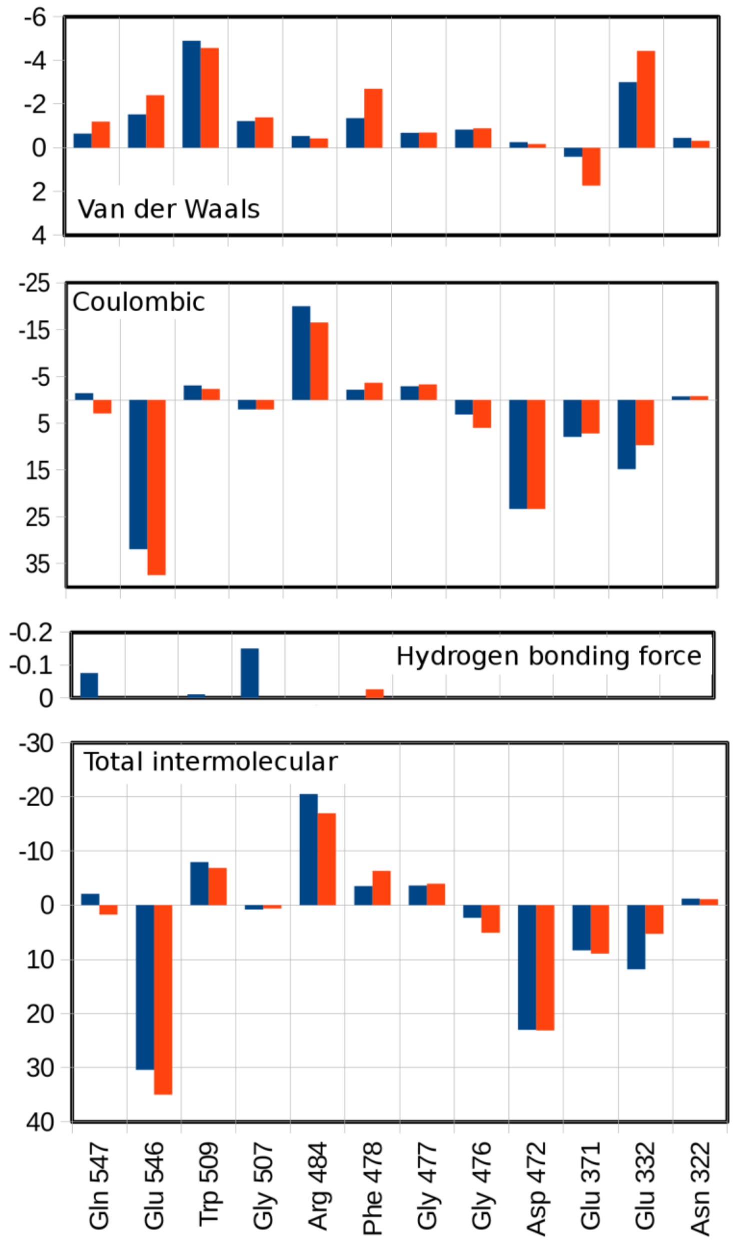

Figure S12. Interaction energies between MurNAc and close amino acid residues in WT-GlcNAcox complex determined during calculation of binding Glice scores for best binding acceptors with Schrödinger software. **Red** columns correspond to the orientation with C-6 close to GlcNAc oxazoline, **blue** columns correspond to the docking in the unproductive site 2. Negative value means favorable interaction, positive value means unfavorable for binding.

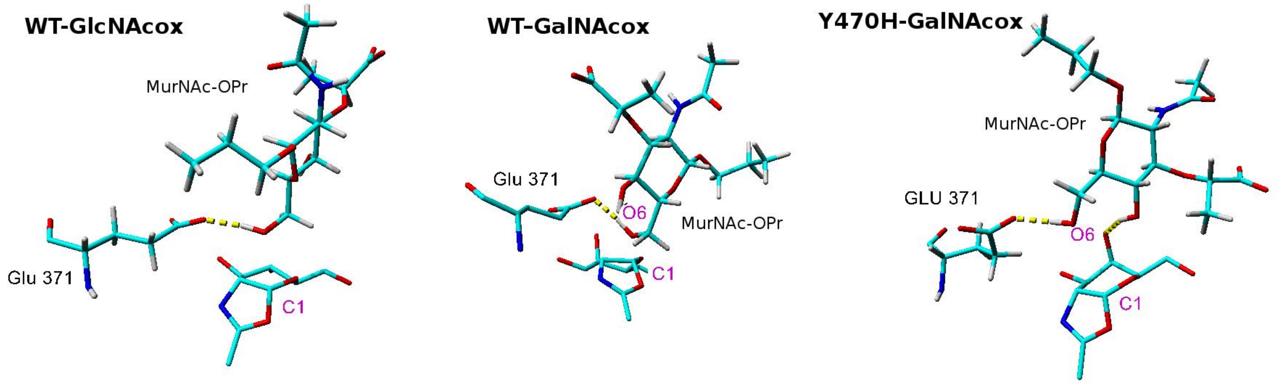

Figure S13. Docked orientation of MurNAc-OPr in the aglycon binding site in WT-GlcNAcox, WT-GalNAcox, and Y470H-GalNAcox complexes.

4. Structural characterization of compounds

Table S1. ^{1}H and ^{13}C NMR data of compound **8** (600.23 MHz for ^{1}H, 150.93 MHz for ^{13}C, D_2O, 30 ˚C)

a) alpha anomer

	**Atom**	δ_C	**m.**	δ_H	**n$_H$**	**m.**	***J*[Hz]**
**GalNAc^A**	**1**	91.33	D	5.261	1	d	3.8
	**2**	50.54	D	4.172	1	dd	11.1, 3.8
	**3**	67.53	D	3.955	1	dd	11.1, 3.3
	**4**	68.74	D	4.024	1	d	3.3
	**5**	69.44	D	4.235	1	m	
	**6**	69.34	T	4.059	1	dd	11.2, 5.0
				3.83^H	1	m	
	**2-CO**	174.98	S		0		
	**Ac**	22.52^a	Q	2.094	3	s	
**GalNAc^D**	**1**	102.34	D	4.557	1	d	8.5
	**2**	52.67	D	3.955	1	m	
	**3**	71.24	D	3.78^H	1	m	
	**4**	68.08	D	3.989	1	d	3.3
	**5**	75.42	D	3.73^H	1	m	
	**6**	61.28	T	3.84^H	2	m	
	**2-CO**	175.20	S		0		
	**Ac**	22.50	Q	2.096	3	s	

b) beta anomer

	**Atom**	δ_C	**m.**	δ_H	**n$_H$**	**m.**	***J*[Hz]**
**GalNAc^A**	**1**	95.69	D	4.671	1	d	8.5
	**2**	54.05	D	3.906	1	dd	10.8, 8.5
	**3**	71.24	D	3.755^T	1	m	
	**4**	68.07	D	3.96^H	1	m	
	**5**	74.12	D	3.81^H	1	m	

	**6**	69.29	T	4.073	1	dd	11.0, 4.5
				3.87[H]	1	m	
	**2-CO**	175.28	S		0		
	**Ac**	22.26[a]	Q	2.098	3	s	
**GalNAc[D]**	**1**	102.45	D	4.561	1	d	8.5
	**2**	52.67	D	3.955	1	m	
	**3**	71.24	D	3.78[H]	1	m	
	**4**	68.08	D	3.989	1	d	3.3
	**5**	75.39	D	3.73[H]	1	m	
	**6**	61.28	T	3.84[H]	2	m	
	**2-CO**	175.18	S		0		
	**Ac**	22.50	Q	2.096	3	s	

[A] … acceptor GalNAc unit (non-reducing end); [D] … donor GalNAc unit (reducing end)
[H] … HSQC readout; [a] … might be interchanged

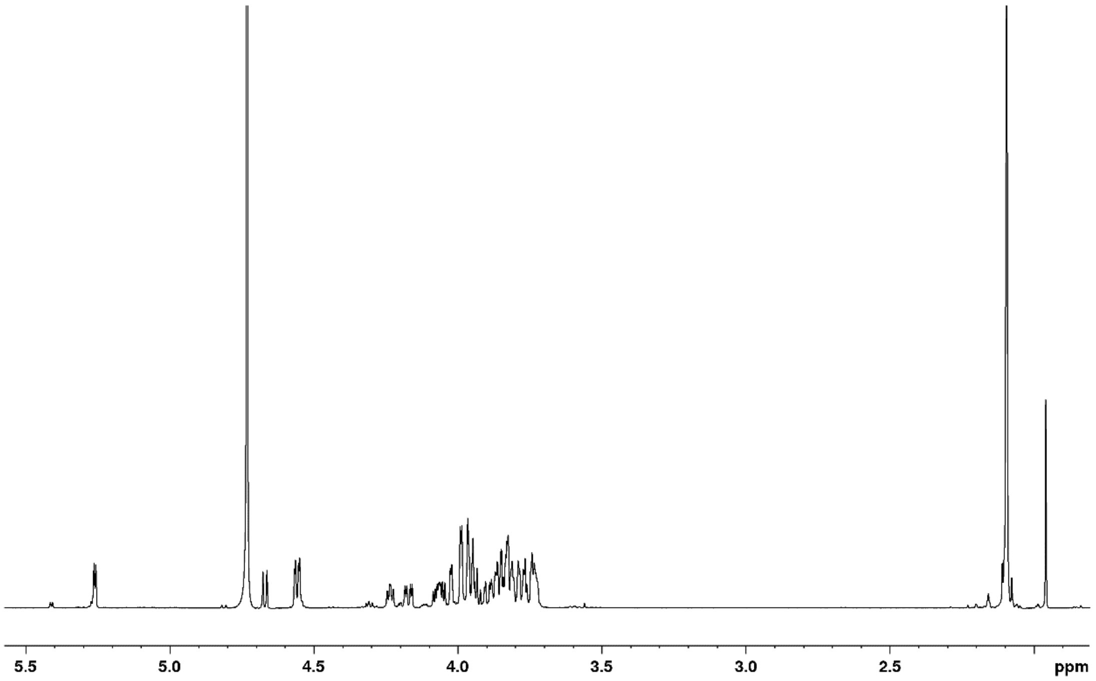

Figure S14a. ^{1}H NMR spectrum of both anomers of compound **8** (600.23 MHz for ^{1}H, D_2O, 30 °C).

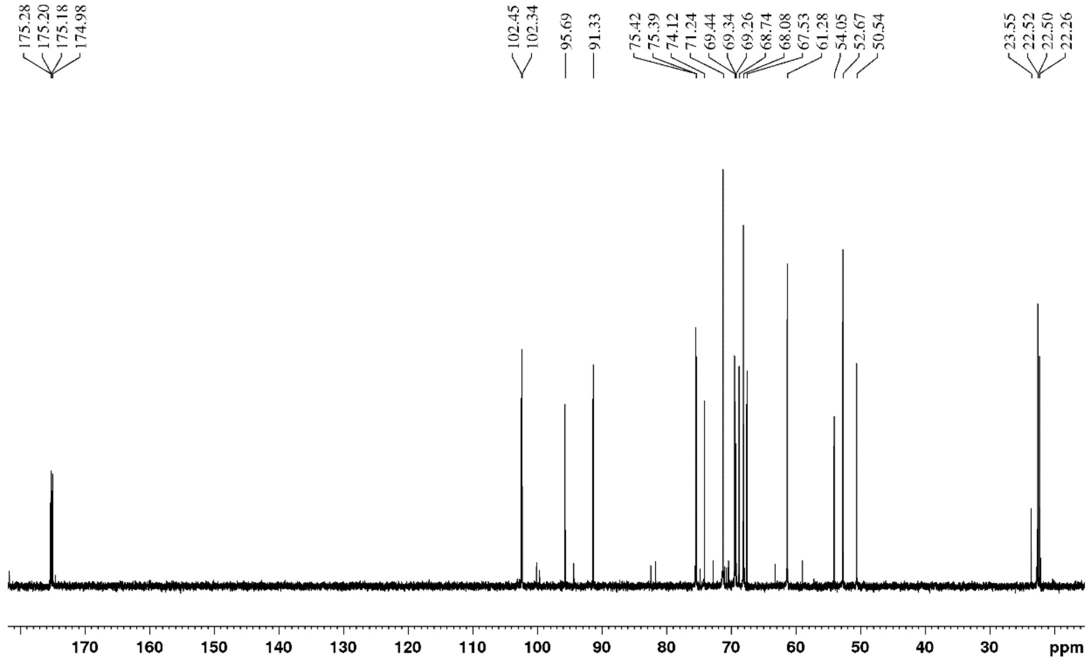

Figure S14b. ^{13}C NMR spectrum of both anomers of compound **8** (150.93 MHz for ^{13}C, D_2O, 30 ˚C)

Table S2. ^{1}H and ^{13}C NMR data of compound **9** (700.13 MHz for ^{1}H, 176.05 MHz for ^{13}C, D_2O, 30 ˚C)

	**Atom**	δ_C	**m.**	δ_H	**n**$_H$	**m.**	***J*[Hz]**
**Glc**	**1**	99.37	D	4.765	1	d	8.1
	**2**	72.83	D	3.298	1	dd	9.5, 8.1
	**3**	75.85	D	3.501	1	dd	9.5, 9.0
	**4**	69.94	D	3.375	1	dd	9.8, 9.0
	**5**	76.44	D	3.469	1	m	
	**6**	61.20	T	3.94^{H}	1	m	
				3.72^{H}	1	m	
**GlcNAc**	**1**	98.11	D	4.890	1	d	8.6
	**2**	55.52	D	3.763	1	dd	10.4, 8.6
	**3**	74.14	D	3.577	1	m	
	**4**	70.07	D	3.481	1	m	
	**5**	76.31	D	3.481	1	m	
	**6**	60.9^{H}	T	3.93^{H}	1	m	
				3.76^{H}	1	m	
	**2-CO**	175.24	S	-	0	-	
	**Ac**	22.5^{H}	Q	2.051	3	s	

^{H} … signal was not extracted - HSQC readout

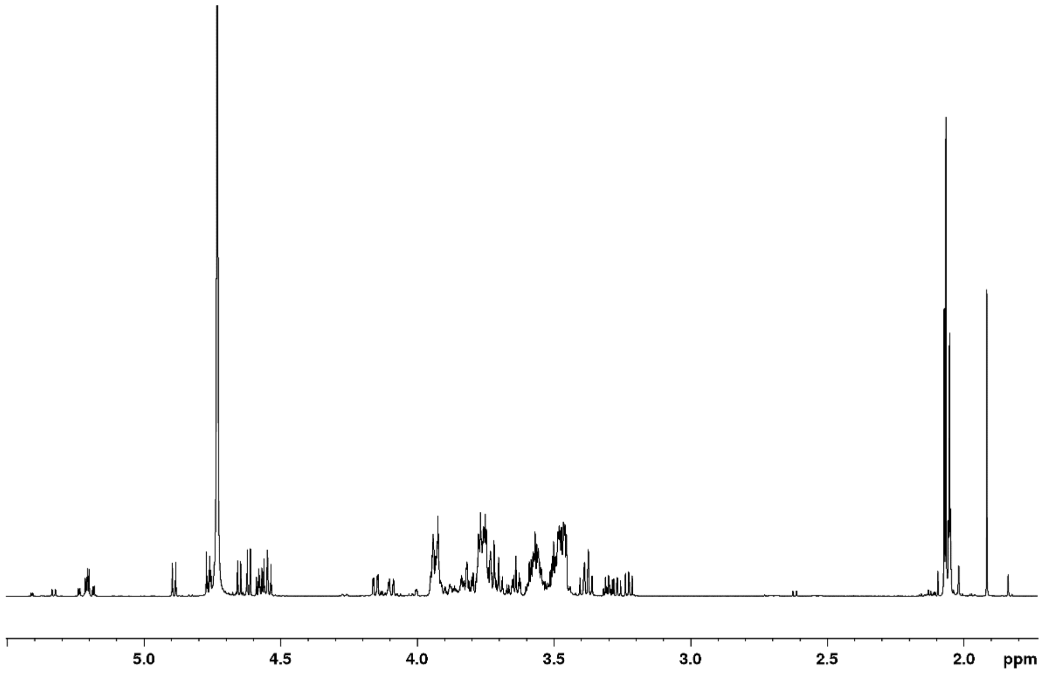

Figure S15a. ^{1}H NMR spectrum of unpure compound **9** (700.13 MHz for ^{1}H, D_2O, 30 ˚C).

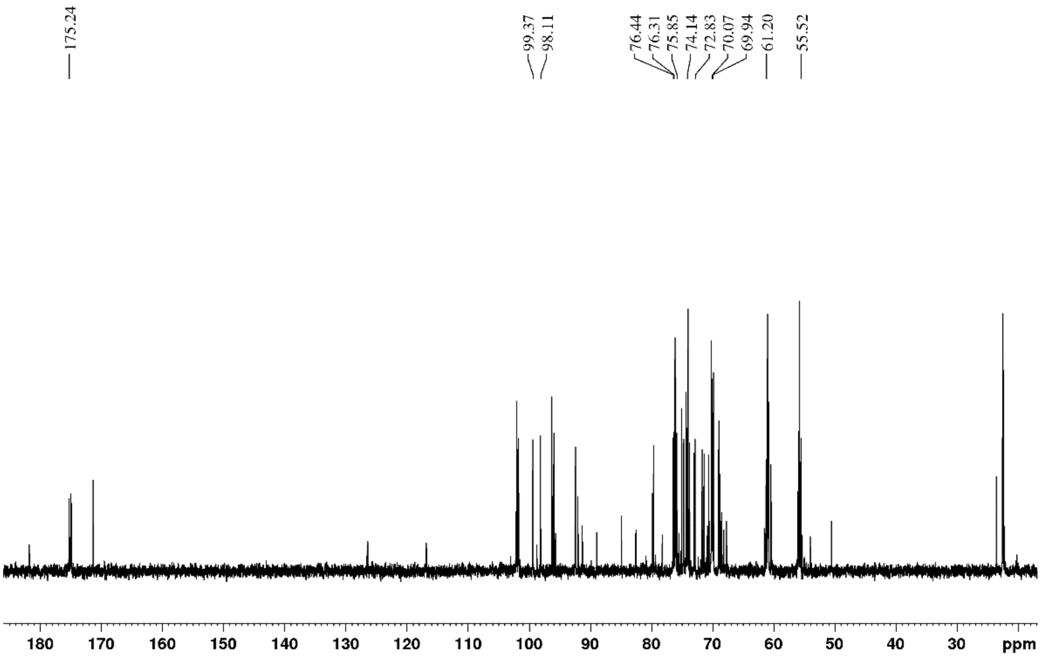

Figure S15b. ^{13}C NMR spectrum of unpure compound **9** (176.05 MHz for ^{1}H, D_2O, 30 ˚C). Only peaks belonging to compound **9** are labelled.

Table S3. ^{1}H and ^{13}C NMR data of compound **10** (600.23 MHz for ^{1}H, 150.93 MHz for ^{13}C, D_2O, 30 ˚C)

	Atom	δ_C	m.	δ_H	n_H	m.	[Hz]

**Gal**	**1**	100.07	D	4.738	1	d	8.0
	**2**	70.53	D	3.571	1	dd	9.9, 8.0
	**3**	72.90	D	3.700	1	dd	9.9, 3.4
	**4**	68.91	D	3.967	1	m	
	**5**	75.57	D	3.73[H]	1	m	
	**6**	61.37	T	3.82[H]	2	m	
**GlcNAc**	**1**	98.26	D	4.492	1	d	8.6
	**2**	55.59	D	3.810	1	dd	10.4, 8.6
	**3**	74.15	D	3.623	1	m	
	**4**	70.12	D	3.525	1	m	
	**5**	76.30	D	3.525	1	m	
	**6**	60.95	T	3.98[H]	1	m	
				3.81[H]	1	m	
	**2-CO**	175.29	S	-	0	-	
	**Ac**	22.57	Q	2.099	3	s	

[H] … HSQC readout

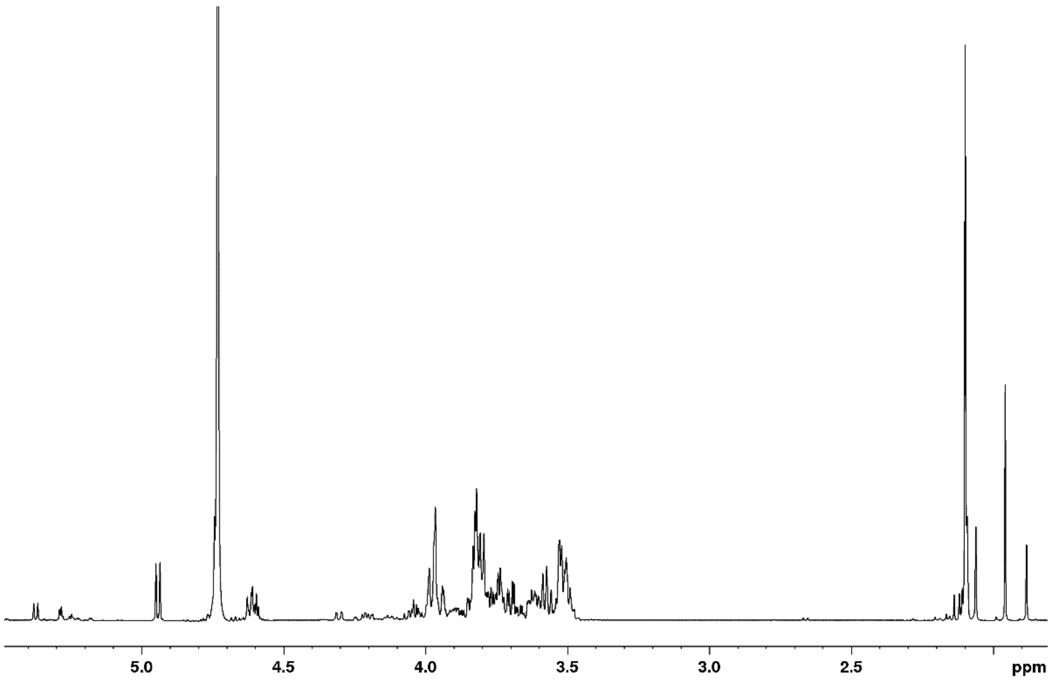

Figure S16a. ^{1}H NMR spectrum of compound **10** (600.23 MHz for ^{1}H, D_2O, 30 °C).

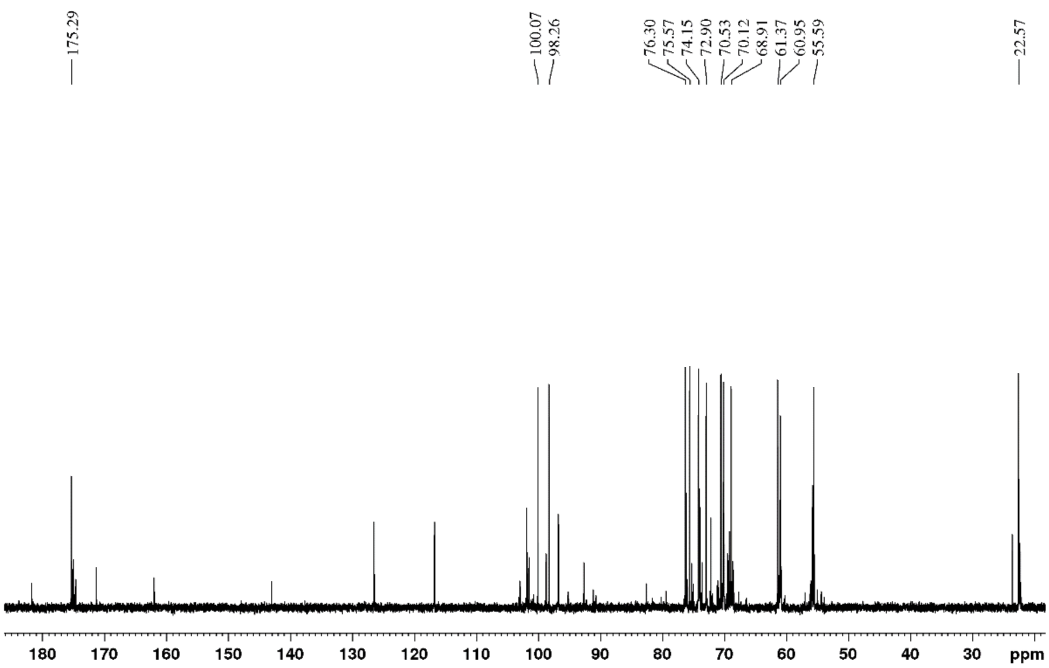

Figure S16b. ^{13}C NMR spectrum of compound **10** (150.93 MHz for ^{13}C, D_2O, 30 °C).

Table S4. ^{1}H and ^{13}C NMR data of compound **16** (700.13 MHz for ^{1}H, 176.05 MHz for ^{13}C, D_2O, 30 °C)

	**Atom**	δ_C	**m.**	δ_H	n_H	**m.**	**J[Hz]**
**spacer**	**1′**	72.42	T	3.804	1	ddd	10.0, Σ*J* = 12.5
				3.552	1	ddd	10.0, Σ*J* = 13.0
	**2′**	22.31	T	1.552	2	m	
	**3′**	9.88	Q	0.873	3	t	7.4
**Mur**	**1**	101.56	D	4.498	1	d	8.5
	**2**	54.59	D	3.672	1	dd	10.5, 8.5
	**3**	81.04	D	3.561	1	dd	10.5, 8.9
	**4**	69.58	D	3.453	1	dd	9.9, 8.9
	**5**	74.74	D	3.574	1	ddd	9.9, 6.0, 1.9
	**6**	68.81	T	4.217	1	dd	11.4, 1.9
				3.737	1	dd	11.4, 6.0
	**2-CO**	174.60	S	-	0		
	**2-Ac**	22.55	Q	2.024	3	s	
	**1″**	77.53	D	4.239	1	q	6.9
	**CH_3**	19.04	Q	1.337	3	d	6.9
	**2″**	180.76	S	-	0		
**GalNAc**	**1**	102.24	D	4.485	1	d	8.5
	**2**	52.64	D	3.931	1	dd	10.8, 8.5
	**3**	71.23	D	3.747	1	dd	10.8, 3.3
	**4**	68.13	D	3.950	1	dd	3.3, 1.0
	**5**	75.38	D	3.693	1	ddd	7.9, 4.5, 1.0

	**6**	61.26	T	3.830	1	dd	11.7, 7.9
				3.779	1	dd	11.7, 4.5
	**2-CO**	174.96	S	-	0		
	**2-Ac**	22.59	Q	2.060	3	s	

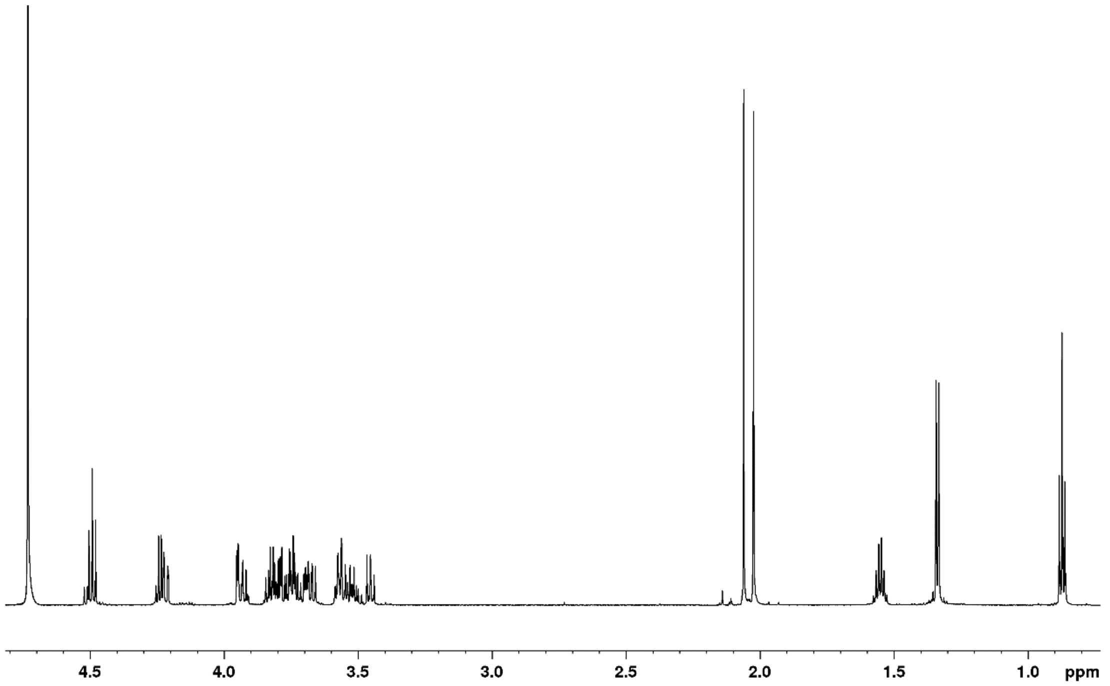

Figure S17a. ^{1}H NMR spectrum of compound **16** (700.13 MHz for ^{1}H, D_2O, 30 °C).

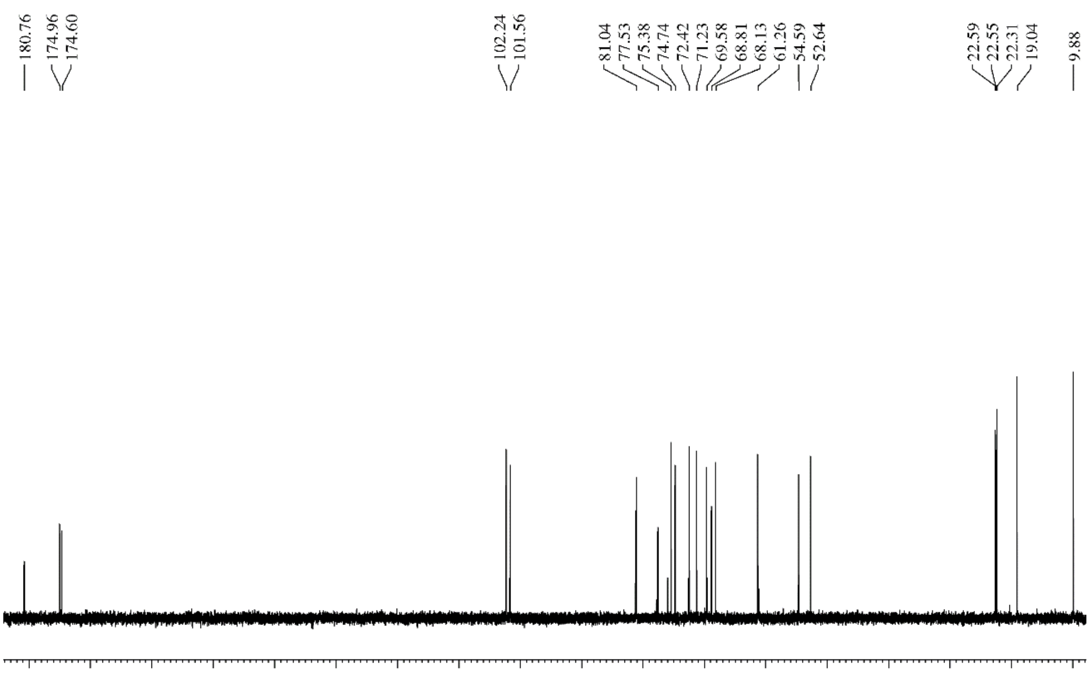

Figure S17b. ^{13}C NMR spectrum of compound **16** (176.05 MHz for ^{13}C, D_2O, 30 °C).

5. Abbreviations

Gal	Galactose
GalNAc	*N*-Acetylgalactosamine
GalNAcox	*N*-Acetylgalactosamine oxazoline (catalytic intermediate during enzymatic hydrolysis)
Glc	Glucose
GlcA	Glucuronic acid
GlcNAc	*N*-acetylglucosamine
GalNAcox	*N*-Acetylglucosamine oxazoline (catalytic intermediate during enzymatic hydrolysis)
HB	Hydrogen bond
MurNAc	*N*-acetylmuramic acid
MurNAc-*O*Pr	Propyl glycoside of *N*-acetylmuramic acid
*p*NP-GalNAc	4-Nitrophenyl *N*-acetyl-β-D-galactosaminide
*p*NP-GlcNAc	4-Nitrophenyl *N*-acetyl-β-D-glucosaminide
*Tf*Hex WT	Wild type β-*N*-acetylhexosaminidase from *Talaromyces flavus*
*Tf*Hex Y470H	Mutant β-*N*-acetylhexosaminidase from *Talaromyces flavus*, Tyr470 is exchanged for His
RMSD	Root means square deviation
